# The Fitness Landscape of HIV-1 Gag: Advanced Modeling Approaches and Validation of Model Predictions by *In Vitro* Testing

**DOI:** 10.1371/journal.pcbi.1003776

**Published:** 2014-08-07

**Authors:** Jaclyn K. Mann, John P. Barton, Andrew L. Ferguson, Saleha Omarjee, Bruce D. Walker, Arup Chakraborty, Thumbi Ndung'u

**Affiliations:** 1 HIV Pathogenesis Programme, Doris Duke Medical Research Institute, Nelson R. Mandela School of Medicine, University of KwaZulu-Natal, Durban, South Africa; 2 KwaZulu-Natal Research Institute for Tuberculosis and HIV (K-RITH), Nelson R Mandela School of Medicine, University of KwaZulu-Natal, Durban, South Africa; 3 Department of Chemical Engineering, Massachusetts Institute of Technology, Cambridge, Massachusetts, United States of America; 4 Ragon Institute of Massachusetts General Hospital, Massachusetts Institute of Technology and Harvard University, Boston, Massachusetts, United States of America; 5 Department of Materials Science and Engineering, University of Illinois at Urbana-Champaign, Urbana, Illinois, United States of America; 6 Howard Hughes Medical Institute, Chevy Chase, Maryland, United States of America; 7 Departments of Chemistry and Physics, Massachusetts Institute of Technology, Boston, Massachusetts, United States of America; 8 Max Planck Institute for Infection Biology, Berlin, Germany; ETH Zurich, Switzerland

## Abstract

Viral immune evasion by sequence variation is a major hindrance to HIV-1 vaccine design. To address this challenge, our group has developed a computational model, rooted in physics, that aims to predict the fitness landscape of HIV-1 proteins in order to design vaccine immunogens that lead to impaired viral fitness, thus blocking viable escape routes. Here, we advance the computational models to address previous limitations, and directly test model predictions against *in vitro* fitness measurements of HIV-1 strains containing multiple Gag mutations. We incorporated regularization into the model fitting procedure to address finite sampling. Further, we developed a model that accounts for the specific identity of mutant amino acids (Potts model), generalizing our previous approach (Ising model) that is unable to distinguish between different mutant amino acids. Gag mutation combinations (17 pairs, 1 triple and 25 single mutations within these) predicted to be either harmful to HIV-1 viability or fitness-neutral were introduced into HIV-1 NL4-3 by site-directed mutagenesis and replication capacities of these mutants were assayed *in vitro*. The predicted and measured fitness of the corresponding mutants for the original Ising model (*r* = −0.74, *p* = 3.6×10^−6^) are strongly correlated, and this was further strengthened in the regularized Ising model (*r* = −0.83, *p* = 3.7×10^−12^). Performance of the Potts model (*r* = −0.73, *p* = 9.7×10^−9^) was similar to that of the Ising model, indicating that the binary approximation is sufficient for capturing fitness effects of common mutants at sites of low amino acid diversity. However, we show that the Potts model is expected to improve predictive power for more variable proteins. Overall, our results support the ability of the computational models to robustly predict the relative fitness of mutant viral strains, and indicate the potential value of this approach for understanding viral immune evasion, and harnessing this knowledge for immunogen design.

## Author Summary

At least 70 million people have been infected with HIV since the beginning of the epidemic and an effective vaccine remains elusive. The high mutation rate and diversity of HIV strains enables the virus to effectively evade host immune responses, presenting a significant challenge for HIV vaccine design. We have developed an approach to translate clinical databases of HIV sequences into mathematical models quantifying the capacity of the virus to replicate as a function of mutations within its genome. We have previously shown how such “fitness landscapes” can be used to guide the design of vaccines to attack vulnerable regions from which it is difficult for the virus to escape by mutation. Here, using new modeling approaches, we have improved on our previous models of HIV fitness landscape by accounting for undersampling of HIV sequences and the specific identity of mutant amino acids. We experimentally tested the accuracy of the improved models to predict the fitness of HIV with multiple mutations in the Gag protein. The experimental data are in strong agreement with model predictions, supporting the value of these models as a novel approach for determining mutational vulnerabilities of HIV-1, which, in turn, can inform vaccine design.

## Introduction

The ideal way to combat the spread of HIV-1 is with an effective prophylactic or therapeutic vaccine [1], [2]. One of the greatest challenges hindering the achievement of this goal is the incredible sequence diversity and mutability of HIV-1 [3], which can limit the effectiveness of the immune response [2], [4].

CD8+ T cells are instrumental in reducing viral load in HIV-1 acute infection [5] and in maintaining the viral set point during chronic HIV/SIV infection [6], [7]. However, HIV-1 is able to escape the CD8+ T cell response through mutations in or adjacent to HIV-1 epitopes that are presented by HLA class I molecules on the surface of the infected cells [7]. One proposed strategy for realizing a potent prophylactic or therapeutic vaccine is to target CD8+ T cell responses to conserved regions of HIV-1, aiming to reduce incidences of immune escape or, if escape occurs, to reduce viral fitness and lower the viral set point, thereby slowing disease course and reducing transmission at the population level [8], [9]. While escape mutations at highly conserved sites often damage the viability of virus [10], this approach is confounded by the development of compensatory mutations which restore or partially restore viral fitness [9]. Thus, to maximize the effectiveness of a vaccine-induced immune response one must look beyond conservation of single residues to identify regions where mutations are not only highly deleterious, but where further mutations elsewhere in the proteome are unlikely to restore lost fitness, but rather, lead to additional fitness costs due to deleterious synergistic effects.

Our group has developed computational models to identify such vulnerable regions of the HIV-1 proteome and to predict the fitness landscape of HIV-1 proteins, providing tools for designing vaccine immunogens that may limit both HIV-1 evasion of CD8+ T cell responses and the development of compensatory mutations [11], [12]. In an early qualitative study we identified groups of amino acids in HIV-1 Gag coupled by structural and functional constraints that cause these residues to co-evolve with each other, but evolve nearly independently of the other residues in the protein [12]. In analogy with past studies on the economic markets and enzymes [13]–[15], we termed these groups of residues “sectors”. This analysis and human clinical data revealed one sector in Gag, which we termed sector 3, where multiple mutations were more likely to be deleterious. This group of residues is naturally targeted more by elite controllers [12]. It is expected to be particularly vulnerable to CD8+ T cell responses that target multiple residues in it since multiple mutations within this sector are likely to significantly diminish viral fitness, thereby restricting available escape and compensatory paths [12].

This approach, however, does not allow us to determine precisely which residues should be targeted, as it does not quantify the relative replicative viability of viral strains bearing specific mutations. Nor does it identify viable escape routes that remain upon targeting residues in the vulnerable regions, or inform how best to block them. To begin to address these issues, we developed a computational model, rooted in statistical physics, which aims to predict the viral fitness landscape (viral fitness as a function of amino acid sequence) from sequence data alone and applied it to HIV-1 Gag [11]. Similar methods have previously been employed to study other complex biological systems, from describing the activity patterns of neuronal networks [16]–[19] to the prediction of contact residues in protein families [17], [20], [21].

The idea underlying our approach is to first characterize the distribution of sequences in the population, which we expect to be correlated with fitness (see below). Due to the small number of available sequences compared to the size of the sequence space, direct estimation of the probability distribution characterizing the available sequences is precluded. Thus, we instead aim to infer the least biased probability distribution of sequences that fits the observed frequency of mutations at each site, and all correlations between pairs of mutations (the one- and two-point mutational probabilities). Mathematically, “least biased” implies the distribution that has maximum entropy in the information-theoretic sense [22]. The maximum entropy distribution that fits the one- and two-point mutational probabilities has a form reminiscent of that describing equilibrium configurations of an Ising model in statistical mechanics. We generated such models using multiple sequence alignments (MSA) for the four subunit proteins of Gag in HIV-1 clade B [11] (described in Supporting Information Text S1, Section 1). This model assigns to each viral strain an “energy” (E), which is inversely related to the probability of observing this sequence.

We expect more prevalent sequences to be more fit, consistent with expectations from simple models of evolution [23] though the precise correspondence between fitness and prevalence may have a more complicated dependence on factors such as the shape of the fitness landscape, as predicted by quasispecies theory [24]. Furthermore, this expectation could be confounded by immune responses in the patients from whom the virus samples were collected, and phylogeny. Recent analyses suggest (described more fully in the discussion) that in spite of these effects, at least for Gag proteins, the rank order of prevalence and *in vitro* replicative fitness should be similar [25]. Strains with high E values are predicted to be less fit than strains with low E values. Predictions of the model seemed to be in good agreement with experimental data on *in vitro* replicative fitness, as well as clinical observations on the frequency and impact of viral escape mutations [11].

Our aim in the current work is twofold. First, we present new advances in the inference and modeling of viral fitness landscapes that address previous theoretical and computational limitations. Second, we describe new *in vitro* fitness measurements for viruses containing multiple Gag mutations, performed to further test fitness predictions using the improved computational methods. To give a broad test of the predictive power of the fitness models, we have performed comparisons for HIV-1 strains containing multiple mutations predicted to harm HIV-1 viability as well as combinations predicted to be relatively fitness neutral. We find that fitness measurements of these mutant strains are in good agreement with model predictions.

## Methods

### Computational models to translate sequence data to viral fitness landscapes

Our key hypothesis in formulating models of HIV fitness is that the prevalence of viruses with a given sequence, that is, how often the sequence is observed, is related to its fitness. Simply, fitter viruses should be more frequent in the population than those that are unfit. This hypothesis can be proven for some idealized evolutionary models [23], but cannot be made exact for the complicated nonequilibrium host-pathogen riposte between humans and HIV. However, our theoretical work, backed by extensive computational studies, suggests that the rank order of fitness and prevalence of strains should be strongly monotonically correlated, provided we compare sequences that are phylogenetically close [25]. Thus, if we construct a model to predict the likelihood of observing different viral strains with given sequences, it can predict the relative fitness of the strains. We achieved this goal by constructing a maximum entropy model for the probability of observing sequences in the MSA [26]. The simplest model in this class is an Ising model, a simple model of interacting binary variables from statistical physics which has been widely applied to study collective behavior in complex systems. The parameters of this Ising model are obtained by imposing the constraint that it reproduce the pattern of correlated mutations (relative to the consensus sequence) observed in a multiple sequence alignment (MSA) of HIV-1 amino acid sequences extracted from infected hosts. Specifically, the parameters were chosen such that the frequency of mutations at each single residue and the frequency of simultaneous mutations at each pair of residues were the same in both the Ising model and the MSA. Importantly, the model also reproduced higher order mutational correlations accurately, even though these mutational frequencies were not directly fitted [11].

As described in our previous publication [11], in the Ising model amino acid sequences in the MSA are compressed into binary strings by assigning a 0 to each position where the amino acid matches the consensus sequence (“wild-type”), and a 1 to each position with a mismatch (“mutant”). While this binary approximation greatly simplified our modeling approach, the reduction in complexity has several drawbacks. Firstly, there is a loss of residue-specific resolution. The fitness predictions of our model are insensitive to the precise identity of mutant amino acids, and thus the model cannot resolve fitness differences between proteins containing different mutant amino acid residues in a particular position. Secondly, for relatively conserved proteins such as HIV-1 Gag, where the number of viable amino acids at each position is rather limited, this binary simplification represents a reasonable approximation. However, it is less justified for highly mutable proteins where the wild-type residue in each position is not the overwhelmingly most probable amino acid, as is the case for the HIV-1 Env protein.

In our original approach, we fit the Ising model parameters to precisely reproduce the observed one and two-residue mutational correlations within the MSA. However, simultaneous mutations at certain pairs of residues were never observed. This led to another deficiency in our original modeling approach in that pairs of mutations not observed in the MSA were predicted to be completely unviable (*E* = ∞). While it is possible that such mutant viral strains have exactly zero replicative fitness, it is more likely that they are highly unfit strains (possessing non-zero replicative fitness) that simply arise too seldom to be observed within our finite-sized MSA.

In this work, we present three significant advances of our original model to predict viral fitness, which also the aforementioned limitations. First, we incorporate Bayesian regularization into our fitting procedure to eliminate the prediction of zero replicative fitnesses for mutations not present within our MSA. Second, we implement a new algorithm for inferring an Ising model from sequence data, which dramatically accelerates the computation of model parameters. Third, we relax the binary approximation to infer viral fitness landscapes that explicitly retain the amino acid identities at each position. We achieve this by describing the viral fitness landscape using a multistate generalization of the Ising model known as the Potts model, another established and well-studied model in statistical physics [27]. We also implement Bayesian regularization into the fitting of the Potts model parameters.

### Model 1: Regularized and computationally fast inference of Ising models of viral fitness

Inference of the parameters of the Ising models, commonly referred to as the inverse Ising problem, is a canonical inverse problem lacking an analytical solution that may be tackled in many ways [16], [17], [19]–[21], [28], [29]. We improve upon our previous techniques described in [11] by incorporating regularization and implementing new inference algorithms, which greatly decrease the computational burden and accelerate model fitting.

To control the effects of undersampling and to improve the predictive power of the inferred fitness models, we incorporate Bayesian regularization into our inference algorithm [18], [19], [30], [31] in the form of a Gaussian prior distribution for the model parameters describing pairwise couplings between residues (see Text S1, Sections 1.3 and 2.5). Regularization of this form is also known as Tikhonov regularization or ridge regression [32]. With this addition, the probability of observing any sequence, including those containing pairs of mutations not observed in the MSA, is nonzero. We have also computed a correction to the energy of each sequence to account for the possible bias that strains near fitness peaks are more likely to be observed than would be expected from their intrinsic fitness when sampled from a finite distribution (see Text S1, Section 3.2).

In an algorithmic advance over our previous fitting procedure, we fit the parameters of our regularized Ising model using the selective cluster expansion algorithm of Cocco and Monasson [18], [19] which identifies clusters of strongly interacting sites and iteratively builds a solution for the whole system by solving the inverse Ising problem for each cluster. With this approach, we cut the CPU time necessary to infer the parameters of the Ising model from roughly 12 years [11] to 5 hours for p24, an improvement by four orders of magnitude. Roughly, we expect algorithm run-time to scale as *O*(*Nn* exp(*n*)), where *N* is the system size (number of amino acids) and *n* is the size of a typical “neighborhood” of strongly interacting sites. For a review and applications of this method see [18], [30]. Complete details of our modeling approach and numerical fitting procedures are provided in the Text S1, Section 1.

### Model 2: Regularized Potts models of viral fitness

An ideal model of viral fitness would be able to capture the full (unknown) distribution of correlated mutations throughout the sequence, and thus reproduce the prevalence of every viral strain. Sequences in the MSA represent a sample of the possible strains of the virus, providing information about the distribution of point mutations, pairs of simultaneous mutations, triplets of simultaneous mutations, and all higher orders. However, since the number of available sequences in the MSA is very small compared to the size of the accessible sequence space, and because mutations at most sites are rare, higher order mutations will be severely undersampled. Thus, following our previous approach we appeal to the maximum entropy principle to seek the simplest possible model capable of reproducing the single site and pair amino acid frequencies [11], [22], for which the problem of undersampling is less severe. From this analysis, the Potts model is the least structured model capable of reproducing the one and two-position frequencies of amino acids observed within the MSA [21].

To introduce the Potts model, we represent the sequence of a particular m-residue protein as a vector, 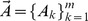, where the elements *A_k_* can take on the *q* = 21 integer values [1, 2, …, 21] denoting an arbitrary encoding of the 20 natural amino acids, plus a gap [21]. In the Potts model the probability 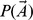 of observing a particular sequence 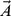 is given by
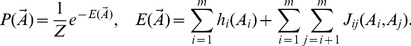
(1)


In analogy with the statistical physics literature, we refer to E as a dimensionless “energy,” the function 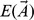 as the Hamiltonian, and the normalizing factor Z as the partition function [27]. The model is parameterized by a set of m q-dimensional vectors, 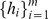, and a set of *m*(*m*−1)/2 q-by-q matrices, 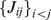. The *h_i_* vectors give the contribution of the identity of each amino acid in each position to the overall sequence energy, and the *J_ij_* matrices give the contribution to the energy of pairwise interactions between amino acids in different positions.

To fit the Potts model, we implemented a generalization of the semi-analytical extension of the iterative gradient descent implemented by Mora and Bialek [11], [17]. This approach implements a multi-dimensional Newton search to iteratively adjust the 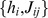 model parameters until the predictions of the model for the one and two-position frequencies of amino acids reproduce those observed within the MSA. In an advance over the original incarnation of this algorithm, we have derived closed form expressions for the gradients required by the Newton search, thereby obviating the need for their numerical estimation by finite differences (which would result in a more computationally expensive and less numerically stable secant search procedure). Our approach is semi-analytical in the sense that while we have analytical expressions for the Newton search gradients, we use a Monte Carlo procedure to numerically estimate the one and two-position amino acid frequencies predicted by the model at each stage of parameter refinement. We are currently developing a Potts generalization of the cluster expansion algorithm [19] to accelerate fitting. We incorporate Bayesian regularization into our fitting procedure in a precisely analogous manner to that described above for the regularized Ising model by introducing a Gaussian prior distribution over the *J_ij_* parameters. Inference of the Potts model parameters for p24 required approximately 1.4 years of CPU time using a generalization of the gradient descent approach described in Ref. [11]. Fitting the model parameters by gradient descent is expected to scale as *O*((*Np*)^2^), where *N* is the number of amino acids in the protein, and *p* is the characteristic number of mutant residues observed at each position. Full details of the fitting and regularization procedures are provided in Text S1, Section 2. The code implementing the inverse Potts inference algorithm is also provided in Supporting Information Code S1.

### *In vitro* experiments

To test the accuracy of these models in predicting the fitness landscape of HIV-1 Gag, we performed *in vitro* experiments to measure the fitness of various Gag mutants. Previously we had measured the *in vitro* replication capacities of 19 Gag p24 mutants, 16 of which contained single mutations in Gag p24, and compared these with fitness predictions of our original Ising model [11]. Here, we extend this work to measure the replication capacities of HIV-1 strains containing various combinations of mutations, predicted to be either harmful to HIV-1 viability or fitness-neutral, in Gag p24 and p17 and we compare measurements not only to the original Ising model described in Ref. [11], but also to regularized versions of Ising and Potts models that we have developed here. Specifically we considered 17 mutations pairs, one triple, and 25 single mutations within these combinations, as listed in Table 1. These mutations were introduced into the widely used laboratory-adapted HIV-1 clade B reference strain NL4-3.

**Table 1:** HIV-1 NL4-3 Gag mutants selected for testing of predicted energy costs (E) by an *in vitro* HIV-1 replication capacity assay.

Mutant	Gag subunit	Category of pairs/triple	E^a^
186I	p24		78.74
269E	p24		43.43
186I269E	p24	Sector 3^b^, high E^c^	Infinity
295E	p24		22.81
186I295E	p24	Sector 3, high E	Infinity
181R	p24		44.62
310T	p24		6.26
181R310T	p24	Sector 3, high E	Infinity
182S	p24		25.13
198V	p24		Infinity
182S198V	p24	Sector 3, high E	Infinity
179G	p24		56.09
229K	p24		44.63
179G229K	p24	Sector 3, high E	97.01
174G	p24		Infinity
243P	p24		66.65
174G243P	p24	Sector 3, high E	Infinity
168I	p24		38.58
315G	p24		19.11
168I315G	p24	HLA-associated, high E	Infinity
331R	p24		11.77
186I331R	p24	HLA-associated, high E	Infinity
302R	p24		11.10
302R315G	p24	HLA-associated, high E	Infinity
315G331R	p24	HLA-associated, high E	Infinity
190I	p24		41.52
190I302R	p24	HLA-associated, high E	Infinity
219Q	p24		6.73
242N	p24		8.68
219Q242N	p24	p24, low E, compensatory	10.80
146P	p24		7.22
147L	p24		3.42
146P147L	p24	p24, low E, compensatory	6.58
326S	p24		4.59
310T326S	p24	p24, low E, sector 3	10.53
173T	p24		5.92
173T286K	p24		4.89
173T286K147L	p24	p24, low E, triple	4.12
12K	p17		3.74
12K54A	p17	p17, low E	4.84
86F	p17		8.00
92M	p17		8.74
86F92M	p17	p17, high E	Infinity

a Energy cost predicted by original Ising model [11] taking into account differences in NL4-3 and the multiple sequence alignment used in model generation. E is 2.98 for wild-type NL4-3 p24 and 3.43 for wild-type NL4-3 p17.

b Mutation pairs within an immunologically vulnerable group of co-evolving residues, termed sector 3, that we previously identified qualitatively [12].

c *E*>90 or *E* = ∞ were considered high E values and *E*<15 were considered low E values.

The tested mutants can be divided into 4 categories, *viz.* (i) Gag p24 pairs with high E values located within a group of co-evolving amino acids termed sector 3 (cf. Ref [12]), (ii) HLA-associated Gag p24 pairs with high E values, (iii) Gag p24 pairs/triple with low E values, and (iv) Gag p17 pairs (Table 1). These mutation combinations were chosen according to E values predicted by the published Ising model [11], where *E*>90 or *E* = ∞ were considered high E values and *E*<15 were considered low E values. Note that, due to the couplings between mutations at different sites, parameterized by the *J_ij_* in equation 1, the E values depend not only on the specific mutations introduced but also on the sequence background. The E values for mutations reported here are computed with the HIV-1 NL4-3 sequence background, which differs from the p17 and p24 MSA consensus sequences by 8 mutations (R15K, K28Q, R30K, K76R, V82I, T84V, E93D, S125N) and 2 mutations (N252H, A340G), respectively. The p24 region of Gag was focused on since this is the most conserved region of the protein. First, we selected six mutation pairs, predicted to be unfavorable in combination, in sector 3 of Gag p24 since we previously found this to be an immunologically vulnerable group of co-evolving residues in which multiple mutations are not well-tolerated [12]. Since it is desirable to identify low fitness/non-viable combinations of escape mutations for vaccine immunogen design aimed at reducing viral fitness or blocking viable escape pathways, we aimed to identify pairs of likely escape mutations with high E values. Virus mutations that are statistically associated with the expression of specific host HLA class I alleles, which also restrict the same epitopes in which the mutations are found, are likely to be CD8+ T cell-driven escape mutations [33]. We therefore tested five high E pairs of mutations located at HLA-associated Gag p24 codons (HLA-associated variants defined in [34], [35]) in or next to optimal CD8+ T cell epitopes (A-list epitopes from the Los Alamos HIV sequence database [36]) that were restricted by the same HLA. For comparison with high E mutation pairs, mutation combinations with low predicted E values were included in testing, comprising known favorable compensatory pairs in Gag p24 where 219Q compensates for the 242N escape mutant [37] and 147L compensates for the 146P escape mutant [10], as well as one pair in sector 3 of Gag p24 and a Gag p24 triple mutant. Additionally, for broader testing, two mutation pairs in Gag p17 were selected. We note that the most commonly observed mutant amino acid at each codon was tested.

We introduced these mutation combinations into the HIV-1 NL4-3 plasmid by site-directed mutagenesis and their presence was confirmed by sequencing, as described previously [38]. Generation of mutant viruses from mutated plasmids and the measurement of their replication capacities were performed as previously [11], [38]. Briefly, mutated plasmids were electroporated into an HIV-1-inducible green fluorescent protein reporter T cell line, harvested at ≈30% infection of cells, and the replication capacities of the resulting mutant viruses were assayed by flow cytometry using the same cell line. Replication capacities were calculated as the exponential slope of increase in percentage infected cells from days 3–6 following infection at a MOI of 0.003, normalized to the growth of wild-type NL4-3 (*RC* = 1). Three independent measurements were taken and averaged. Mutant viruses were re-sequenced to confirm the presence of introduced mutations.

## Results

### Comparison of predictions from different models

The values of E predicted by our original and new modeling approaches for the 43 HIV-1 NL4-3 Gag mutants tested here are shown in Table 2. Absolute comparison of the E values between the models are not meaningful, but the relative E values of mutants are generally in excellent concordance between models (Pearson's correlation, *r*≥0.85 and *p*≤5.3×10^−11^, two-tailed test).

**Table 2:** Energy costs (E) of HIV-1 NL4-3 Gag mutants predicted by computational models.

Mutant	Gag subunit	Ising E^a^	Regularized Ising E^b^	Regularized Potts E^c^
186I	p24	78.74	9.98	11.24
269E	p24	43.43	11.46	12.18
186I269E	p24	Infinity	17.77	18.97
295E	p24	22.81	9.05	11.03
186I295E	p24	Infinity	15.36	17.79
181R	p24	44.62	13.55	12.12
310T	p24	6.26	5.87	7.20
181R310T	p24	Infinity	15.74	14.87
182S	p24	25.13	7.11	9.68
198V	p24	Infinity	12.32	-d
182S198V	p24	Infinity	15.77	-d
179G	p24	56.09	11.14	11.57
229K	p24	44.63	10.52	11.68
179G229K	p24	97.01	17.99	18.81
174G	p24	Infinity	15.47	11.71
243P	p24	66.65	11.1	11.08
174G243P	p24	Infinity	22.9	18.32
168I	p24	38.58	9.8	10.30
315G	p24	19.11	6.85	10.64
168I315G	p24	Infinity	14.78	16.39
331R	p24	11.77	7.37	9.17
186I331R	p24	Infinity	13.68	15.85
302R	p24	11.1	7.75	9.23
302R315G	p24	Infinity	12.4	15.30
315G331R	p24	Infinity	10.56	15.22
190I	p24	41.52	8.2	11.41
190I302R	p24	Infinity	12.28	16.12
219Q	p24	6.73	5.65	6.90
242N	p24	8.68	6.7	8.05
219Q242N	p24	10.8	8.04	10.07
146P	p24	7.22	5.62	6.26
147L	p24	3.42	4.25	6.54
146P147L	p24	6.58	5.77	4.74
326S	p24	4.59	4.78	5.69
310T326S	p24	10.53	7.72	8.81
173T	p24	5.92	5.81	7.02
173T286K	p24	4.89	6.56	7.75
173T286K147L	p24	4.12	5.93	6.78
12K	p17	3.74	1.91	4.38
12K54A	p17	4.84	3.19	5.63
86F	p17	8	4.53	6.00
92M	p17	8.74	6.01	9.43
86F92M	p17	Infinity	9.52	12.57

a E is 2.98 for wild-type NL4-3 p24 and 3.43 for wild-type NL4-3 p17.

b E is 3.67 for wild-type NL4-3 p24 and 1.64 for wild-type NL4-3 p17.

c E is 4.43 for wild-type NL4-3 p24 and 2.81 for wild-type NL4-3 p17.

d The 198V mutation was not observed within the MSA used to fit the Potts model, precluding the fitted model from assigning an energy to viral strains containing this point mutation.

### Experimental findings

**Figure 1:**
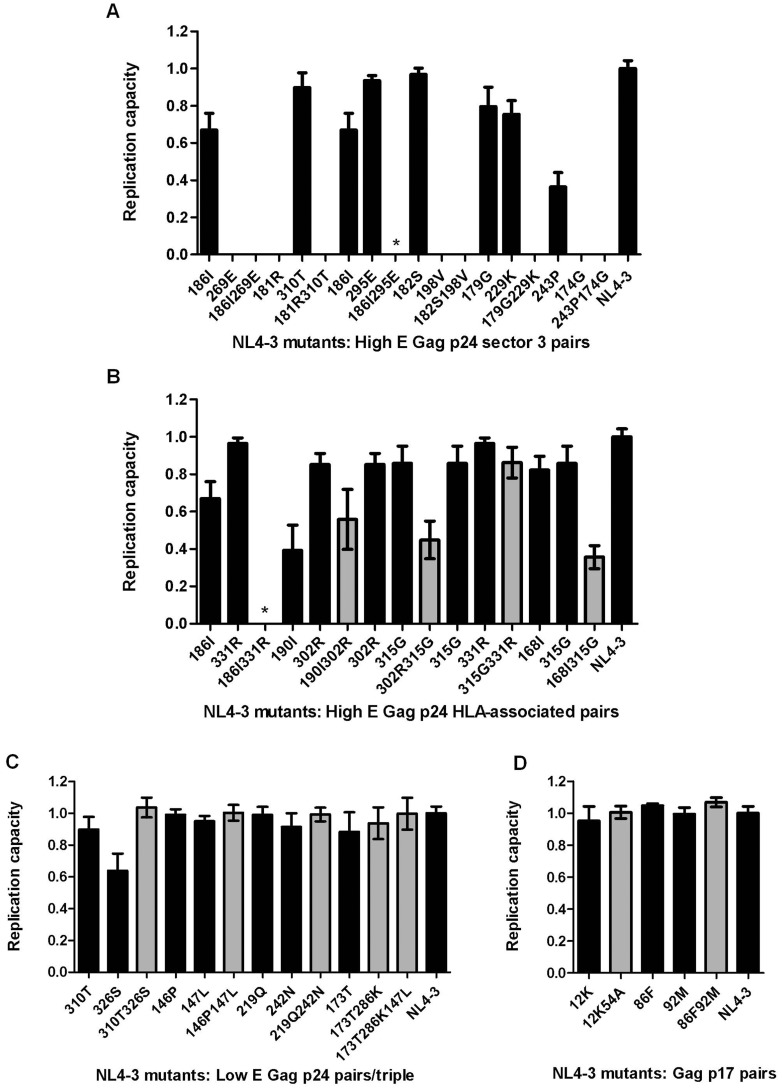
Replication capacities of NL4-3 viruses encoding mutations in HIV-1 Gag. Graphs show replication capacities of NL4-3 viruses encoding (A) Gag p24 mutation pairs with high E values that were previously identified to be in vulnerable co-evolving groups [12] and single mutations within these pairs; (B) Gag p24 HLA-associated pairs with high E values and single mutations within these pairs; (C) Gag p24 pairs/triple with low E values as well as single mutations within these combinations; and (D) Gag p17 pairs including single mutations within the pairs. Those mutants that (i) were not viable or (ii) were not viable unless further mutations developed (indicated with an asterisk), were assigned a replication capacity of zero. Mutation pairs and triples are shown in grey while single mutations within these combinations are shown in black. Replication capacities of mutant viruses are expressed relative to the replication capacity of wild-type NL4-3 virus (*RC* = 1). Bars represent the mean of three independent experiments and error bars represent standard deviation from the mean.

The *in vitro* fitness measurements for all mutants, grouped according to categories, are shown in Figure 1. We initially compared our model predictions and fitness measurements for each category of mutant pairs to evaluate whether mutant combinations with high and low predicted E values corresponded to substantial fitness cost or little/no fitness cost, respectively.

Briefly, all Gag p24 sector 3 mutation pairs with high E values were not viable in our assay system, and were assigned a replication capacity of zero (Figure 1A). Similarly, with the exception of 315G331R, the five high E HLA-associated mutation pairs showed substantial reduction in replication capacity, to between 0–56% of wild-type levels (Figure 1B). Non-viable mutants (*RC* = 0) were those for which the generation of virus stocks from plasmids encoding these mutation pairs failed, or, in two instances – mutants 186I295E and 186I331R – were not viable unless further mutations developed, confirming unfavorability of the mutation combination. Briefly, concentrated virus stocks for mutants 186I295E and 186I331R were harvested at >22 days post-electroporation compared with the median harvesting time of 6 days post-electroporation for all mutants (at which time the 186I295E and 186I331R mutants had infected ≈1% cells). Sequencing of these viruses revealed the presence of additional mutations and/or reversion of introduced mutations. For mutant 186I295E, amino acid mixtures were detected at codons 63 (Q/R), 177 (D/E) and 186 (I/V), and for mutant 186I331R, mixtures were detected at codons 168 (I/V) and 331 (K/R), as well as reversion of 186I to 186T. On repeating virus generation for these mutants, additional mutations similarly developed – mixtures were observed at codons 214 (R/K) and 271 (N/S) for mutant 186I295E, and 232 (R/M) and 260 (D/E) for mutant 186I331R. With the exception of 186I295E and 186I331R, sequencing confirmed that all mutant viruses had only the specific mutations introduced. The spontaneous mutations 186V, 271S and 232M were not observed in the MSA and the new mutation combinations did not have lowered E values in any of the models, with the exception of the incomplete 186I331R260D combination (complete observed combination 186I, 331R, 232R/M, 260D/E) which displayed a slightly lower energy than 186I331R in the regularized Ising model only (11.5 vs. 13.7) (data not shown). Nevertheless, these observations confirm that 186I295E and 186I331R are unfit mutation combinations requiring compensatory paths to restore viability. Taken together, the data on high E p24 mutants confirm mutation combinations predicted to be unfit, and also identify combinations of HLA-associated mutations in/next to optimal CD8+ T cell epitopes (mutations likely to result in CD8+ T cell escape [33]) that carry substantial fitness costs.

Those p24 mutation combinations, including known compensatory pairs, that were predicted to have low E values displayed replication capacities similar to that of wild-type NL4-3, indicating that these combinations had little or no cost to HIV-1 replication capacity in accordance with predictions (Figure 1C). Similarly, all p17 mutants tested had replication capacities close to that of the wild-type NL4-3 virus, consistent with the predicted E values of all mutants except 86F92M (Figure 1D).

Overall, for only two (86F92M and 315G331R) of the 17 mutant pairs the fitness measurement did not correspond to the E value prediction of high or low fitness cost. It should however be noted that the disparity between E values and measured replication capacities for these mutant pairs is somewhat mitigated in the regularized models. The E values for the regularized Ising model for these mutants (which were assigned an E value of infinity by the original Ising model) are lower than those of other mutants previously assigned infinite energies, and the same is true for mutant 86F92M in the regularized Potts model.

### Quantitative comparison between *in silico* predictions and *in vitro* measurements

**Figure 2:**
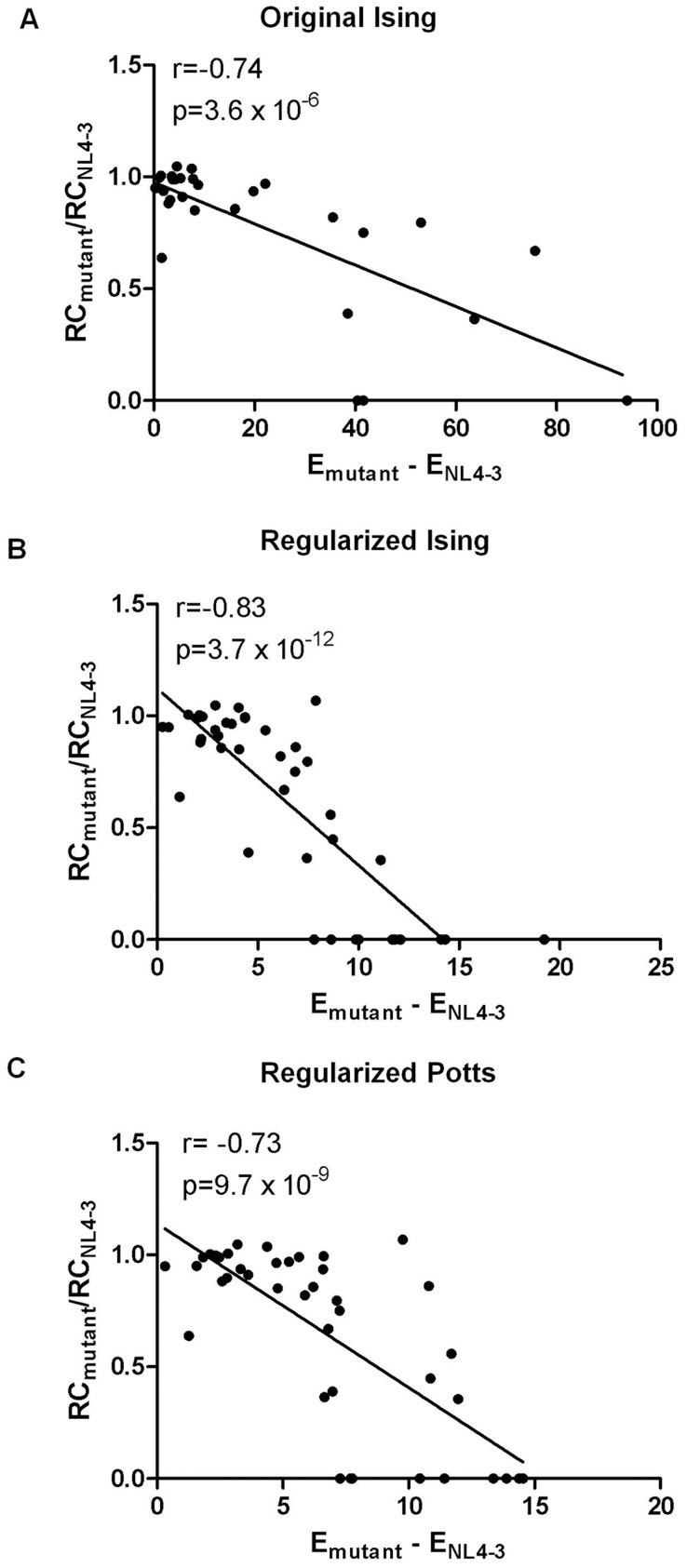
Relationship between predicted E values and replicative capacities of HIV-1 NL4-3 Gag mutants. Scatter plots showing strong correlations between measured replication capacities of mutants and E values predicted by (A) original Ising (Pearson's correlation, *r* = −0.74 and 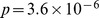, two-tailed test, *n* = 30), (B) regularized Ising (Pearson's correlation, 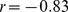 and 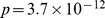, two-tailed test, *n* = 43) and (C) regularized Potts (Pearson's correlation, 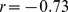 and 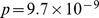, two-tailed test, *n* = 41) models. In the original Ising model (panel A), mutants with E values of infinity (*n* = 13) are excluded from the correlation.

Next, we assessed the relationship between fitness measurements and E values predicted by our original Ising, regularized Ising and regularized Potts models using Pearson's correlation tests. There is a strong correlation between the metric of fitness (values of E, Table 1) predicted by the original unregularized Ising model and our experimental measurements (Pearson's correlation, *r* = −0.74 and *p* = 3.6×10^−6^, two-tailed) (Figure 2A), however this correlation out of necessity excludes mutants with E values equal to infinity (*n* = 13). The regularized Ising model allows for inclusion of these data points resulting in a stronger correlation between predictions and fitness measurements (Pearson's correlation, *r* = −0.83 and *p* = 3.7×10^−12^, two-tailed) (Figure 2B), which is slightly improved by focusing on Gag p24 mutants only (Pearson's correlation, *r* = −0.85 and *p* = 1.4×10^−11^, two-tailed). There is also a strong agreement between the residue-specific Potts model energies and replication capacity (Pearson's correlation, *r* = −0.73 and *p* = 9.7×10^−9^, two-tailed) (Figure 2C).

**Figure 3:**
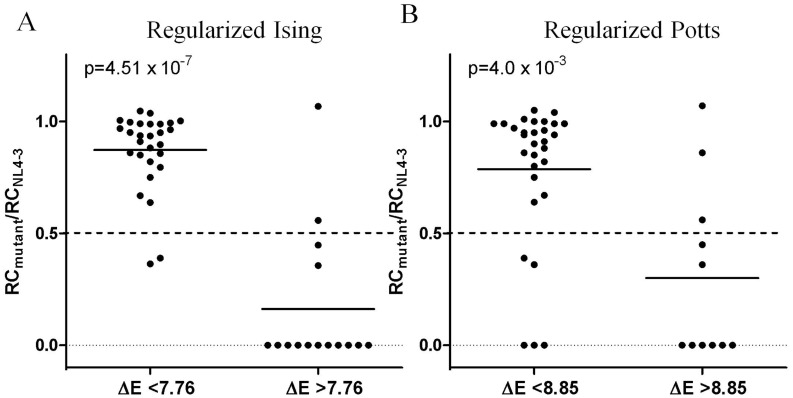
Classification of HIV-1 NL4-3 Gag mutants as unfit/fit using predicted E values. Graphs show the ability of E classifiers, predicted by regularized Ising (panel A) and Potts (panel B) models, to correctly classify HIV-1 NL4-3 Gag mutants into unfit (*RC*<0.5) and fit (*RC*>0.5) categories. The measured replication capacities of mutants classified as fit or unfit according to their predicted E values were compared with the Mann-Whitney test and p values are shown.

In practice, one may be concerned with a more coarse-grained measure of viral fitness: will a virus with a given sequence be able to replicate with similar efficiency to the wild-type, or will it be significantly impaired? To explore this point, we grouped the experimentally tested mutants into two categories, “fit” (*RC*≥0.5) and unfit (*RC*<0.5), and tested the ability of the fitness landscape models to predict which class each sequence would belong to based on their E values. This was accomplished by fitting a linear classifier to the data using logistic regression (Text S1, Section 3.1). The regularized Ising model E classifier is highly accurate (91% accuracy at optimal threshold, *AUROC* = 0.93) – we observed a strong, significant difference in replication capacities between the mutants classified as unfit and those classified as fit (Mann-Whitney *U* = 32, 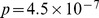) (Figure 3A). Specifically, four mutants (86F92M, 190I, 190I302R and 243P) were not classified correctly. However, 190I302R, which was classified as unfit (*E* = 8.6), exhibited a fitness close to that of the 0.5 cutoff (*RC* = 0.56) and 243P, which displayed low fitness (*RC* = 0.36), had a predicted E value (*E* = 7.4) bordering on the classifier E value. The Potts model classifier also performs well (81% accuracy at optimal threshold, *AUROC* = 0.80), but provides a slightly weaker difference between the fit and unfit classes (Mann-Whitney *U* = 70, 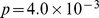) (Figure 3B). Here, seven mutants were not classified correctly, including the same four not classified correctly by the regularized Ising model as well as mutants 174G, 181R, 269E and 315G331R. Similar to mutant 243P, mutants 174G, 181R and 269E were unfit (*RC* = 0) but had a predicted E values ranging from 7.3 to 7.7, fairly close to that of the classifier E value.

## Discussion

In this study, we have substantially advanced our modeling approaches and tested the predictive power of these models by *in vitro* fitness measurements of HIV encoding various mutation combinations in the Gag protein. The *in vitro* functional data are overall in strong agreement with the viral fitness landscape models and support the capacity of these models to robustly predict both continuous and “coarse-grained” measures of HIV-1 *in vitro* replicative fitness. Performance of the regularized Potts and regularized Ising models here is similar, which is not unexpected as Gag in general is not highly mutable and the mutants tested here were the most common ones, making the binary approximation a fairly good assumption. Indeed, in instances where the binary approximation is valid, we might encounter *poorer* performance from the Potts model relative to the Ising due to a diminished ratio of samples (i.e., sequences in the MSA) to parameters (i.e., *h* and *J* values) making robust numerical fitting of the former more challenging than the latter. It is nevertheless encouraging that we are capable of fitting a significantly more complicated Potts model that retains residue-specific resolution without compromising the fidelity of our predictions. Improved inverse Potts inference methods which better meet these numerical challenges may also improve performance of the Potts model with respect to the Ising model results.

Simple theoretical analysis suggests that models which differentiate between different mutant amino acids at the same site, like the Potts model employed here, will be necessary to make fitness predictions for highly mutable proteins such as Env and Nef, or to predict the fitness of sequences containing sites with mutations to less frequently observed amino acids. Using a simple toy model, we show in Text S1, Section 3.4 that the binary approximation (Ising model) has several potential deficiencies compared to a Potts model. In particular, the Ising model generically overestimates the fitness of mutant sequences, particularly for sequences containing uncommon mutations. Also, in the Ising case the inferred interaction between mutations at different sites is dominated by the interaction between the most common mutants, while the Potts model is able to accurately capture interactions between rare mutants. Future work will involve testing Ising and Potts model predictions for more highly variable proteins and for mutations to uncommon amino acids.

While this study confirms the usefulness of this method for predicting HIV-1 replicative fitness, at least for closely related sequences, caution will be necessary in applying this method to predict the relative fitness of multiple strains separated by a large number of mutations. In the measure of prevalence used to infer the Ising and Potts model fitness landscapes, factors such as phylogeny are implicitly included. Analysis conducted in [25] suggests that phylogenetic effects influence the value of the inferred fields h_i_, and that a correction should be included for predictions of energy or fitness. This form of a correction is sensible, as phylogenetic effects should make mutations at individual residues less frequent, leading to larger inferred fields. For closely related strains such as those studied here experimentally, any systematic inaccuracies in the energy due to phylogeny should be similar in magnitude, and thus *differences* in energy should predict relative replicative fitness fairly accurately. This would not necessarily be true, however, for sequences separated by many mutations. Further theoretical developments may be needed to separate out the contributions of phylogeny and intrinsic fitness from the Ising and Potts model landscapes presented here [25] to predict the relative fitness of strains that differ by many mutations.

In addition to phylogeny, other factors such as host-pathogen interactions and pure stochastic fluctuations affect the observed distribution of sequences, and could complicate fitness predictions. In another work [25] we have investigated these issues by carrying out stochastic simulations that aim to mimic the way the samples were collected and host-pathogen dynamics. In this paper, for the p17 protein, we found that the fitness and prevalence were not the same. However, the rank order of fitness and prevalence were the same as long as the strains being compared were not very far apart in sequence space. This is largely because of the diversity of immune responses due to diverse HLA types in the human population. Additionally, the number of virus particles in single infected individuals from whom the virus sequences were extracted is large, as is the number of patients from whom the virus samples were taken. We find that the one and two-point mutational probabilities in the sequence databases have converged [11]; i.e., these correlations do not change upon removal of some sequences, suppressing the effects fluctuations on the inferred model.

We also note that some caution should be taken in comparing E values for sequences belonging to different proteins. The fitness predictions of the Ising and Potts models are unchanged by a constant shift in energy for all sequences, thus comparisons of absolute energy values are not physically meaningful. Differences in energy between two sequences in the same protein, however, can be unambiguously interpreted as the fitness ratio of those sequences. This is the approach we have taken when examining E values from sequences with mutations in p17 and p24 together: rather than comparing the absolute energies, we compare the differences in energy between the mutant and the NL4-3 reference sequence in each protein, which reflect the fitness of the mutant relative to the NL4-3 reference sequence. Finally, translation from differences in energy to differences in fitness might depend on the specific protein that is being considered. While comparisons of energy differences and relative fitnesses of p17 and p24 mutants performed here exhibit no obvious incongruences, further study is needed to confirm the generality of fitness predictions across proteins.

In the case of two mutant pairs (186I295E and 186I331R) that were predicted by the models to have very low fitness, partial reversions and/or additional mutations spontaneously arose in culture that restored virus viability. However, with the exception of one of the spontaneous mutations (260D) observed in combination with 186I331R that modestly decreased the predicted energy (increased fitness) in the regularized Ising model but not the Potts or original Ising models, the models do not predict lower energies (increased fitness) for these mutant pairs in combination with the additional mutations arising *in vitro*. Further, three of the spontaneous mutations – 186V, 271S and 232M – were not observed in the MSA and therefore could not be assessed by the Potts model. As a possible interpretation of these findings, we suggest that it may be the case that these mutation patterns observed *in vitro* are not typically observed *in vivo*, perhaps since these are infrequently explored mutational routes. As a corollary, this could indicate an inherent limitation of computational models derived from clinical sequence data to identify all possible escape-compensatory pathways, and the importance of *in vitro* and *in vivo* experiments to validate and complement model predictions. A mitigating factor, of course, is that mutational pathways observed *in vitro* but not *in vivo* may be of less direct clinical relevance.

In future work, the model predictions will be further validated in animal models by testing the viable escape pathways predicted to emerge following immunization with immunogens containing vulnerable HIV-1 regions only. The validated fitness landscape could then be used to design vaccine immunogens containing epitopes from the vulnerable regions that could be presented by people with diverse HLAs and that target residues particularly harmful to HIV-1 when mutated simultaneously, thereby substantially diminishing viral fitness and/or blocking viable mutational escape [11], [12]. Such immunogens potentially represent good therapeutic vaccine candidates to overcome the challenge of HIV-1 evasion of CD8+ T cell responses. However, further work will also be required to optimize design of such immunogens to ensure that epitopes included are processed effectively and that they are sufficiently immunogenic, as well as to test their immunogenicity, optimal delivery methods and protection efficacy in animal models. Furthermore, fitness landscapes of HIV-1 proteins may also be more widely applied to identify effective antibody targets, and help design potent combinations of neutralizing antibodies for passive immunization as well as small molecule inhibitors for therapy.

## Supporting Information

Code S1 **Source code for the inverse Potts algorithm.** A compressed file containing source code and instructions for its installation and use, along with a set of test data for verifying proper functioning of the code and auxiliary Matlab scripts for computing correlations from a multiple sequence alignment.
(ZIP)

Text S1 **Supplementary methods and figures.** Details for sequence data processing and inference of the Ising (Section 1) and Potts models (Section 2) are given. Details on comparison with experimental results and comparison between Ising and Potts models are given in Section 3. A brief summary of statistical tests is presented in Section 4.
(PDF)
