# Supplementary figures and images for: The Fitness Landscape of HIV-1 Gag: Advanced Modeling Approaches and Validation of Model Predictions by In Vitro Testing
Source: PLoS Comput Biol. 2014 Aug 7;10(8):e1003776. doi: 10.1371/journal.pcbi.1003776 (PMC4125067; doi:10.1371/journal.pcbi.1003776)

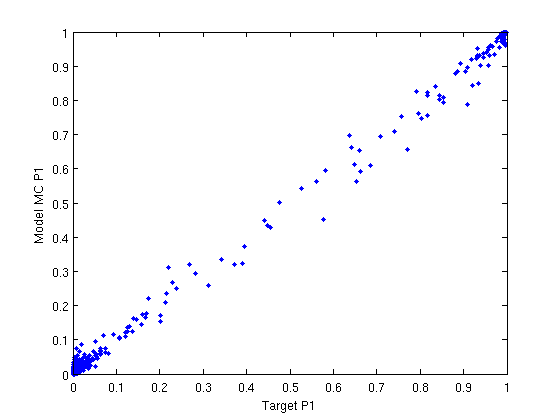

Supplement: Code S1 — Source code for the inverse Potts algorithm. A compressed file containing source code and instructions for its installation and use, along with a set of test data for verifying proper functioning of the code and auxiliary Matlab scripts for computing correlations from a multiple sequence alignment. (ZIP) [file pcbi.1003776.s001.zip › HReconstr_Potts/P1_test.png]

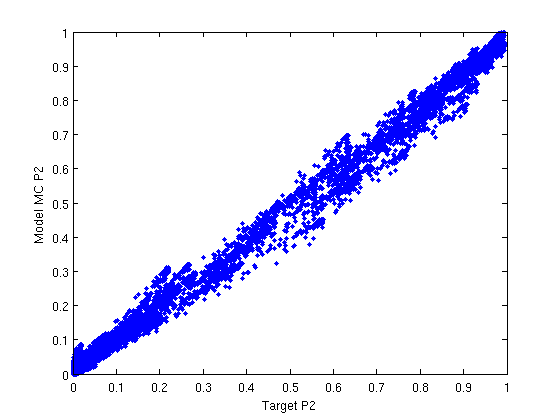

Supplement: Code S1 — Source code for the inverse Potts algorithm. A compressed file containing source code and instructions for its installation and use, along with a set of test data for verifying proper functioning of the code and auxiliary Matlab scripts for computing correlations from a multiple sequence alignment. (ZIP) [file pcbi.1003776.s001.zip › HReconstr_Potts/P2_test.png]
