# Supplementary material for: The Fitness Landscape of HIV-1 Gag: Advanced Modeling Approaches and Validation of Model Predictions by In Vitro Testing
Source: PLoS Comput Biol. 2014 Aug 7;10(8):e1003776. doi: 10.1371/journal.pcbi.1003776 (PMC4125067; doi:10.1371/journal.pcbi.1003776)
Supplement: Text S1 — Supplementary methods and figures. Details for sequence data processing and inference of the Ising (Section 1) and Potts models (Section 2) are given. Details on comparison with experimental results and comparison between Ising and Potts models are given in Section 3. A brief summary of statistical tests is presented in Section 4. (PDF) [file pcbi.1003776.s002.pdf]

SUPPORTING INFORMATION FOR
The Fitness Landscape of HIV-1 Gag

Jaclyn K. Mann[1,2,#], John P. Barton[3,4,&], Andrew L. Ferguson[5,&], Saleha Omarjee[1,2], Bruce D. Walker[1,4,6], Arup K. Chakraborty[3,4,7,*], and Thumbi Ndung'u[1,2,4,8,**]

[1] HIV Pathogenesis Programme, Doris Duke Medical Research Institute, Nelson R. Mandela School of Medicine, University of KwaZulu-Natal, Durban, South Africa.

[2] KwaZulu-Natal Research Institute for Tuberculosis and HIV (K-RITH), Nelson R Mandela School of Medicine, University of KwaZulu-Natal.

[3] Department of Chemical Engineering, Massachusetts Institute of Technology, MA, USA.

[4] Ragon Institute of Massachusetts General Hospital, Massachusetts Institute of Technology and Harvard University, Boston, MA, USA.

[5] Department of Materials Science and Engineering, University of Illinois at Urbana-Champaign, Urbana, Illinois, USA.

[6] Howard Hughes Medical Institute, Chevy Chase, MD, USA.

[7] Departments of Chemistry and Physics, Massachusetts Institute of Technology, Boston, MA, USA.

[#] This author led the experimental work reported in the paper.

[&] These authors led the computational work reported in the paper.

[*] Phone: 617 253 3890. Facsimile: 617 253 2272. Email: arupc@mit.edu.

[**] Phone: +27 31 260 4727; Facsimile +27 31 260 4623; Email: ndungu@ukzn.ac.za.

March 31, 2014

1. Ising model inference

1.1 Data preparation

We downloaded multiple sequence alignments (MSA) for the HIV-1 clade B Gag proteins p17 and p24 from the Los Alamos National Laboratory HIV database (http://www.hiv.lanl.gov). To obtain a represenative sample of the population we selected only one sequence per patient and sequences labeled by the database as "problematic" were excluded, yielding a total of 4389 sequences for p17 and 4003 sequences for p24. After downloading, the MSA data was processed to remove insertions relative to the HXB2 reference sequence (Leitner *et al.*, 2005). The nucleotide sequences were then translated into sequences of amino acids, with ambiguous codons translated as blanks.

We determined the most common amino acid at each position in the proteins, which we refer to as the "wild-type" amino acid. We then translated each sequence in the MSA into a binary form by assigning a 0 to each position where the amino acid matched the wild-type, and a 1 to each position where there was a mismatch. For example, if the wild-type amino acid sequence for a protein segment is MGARAS, a sequence MGAIAS would be written in binary form as 000100. Both p17 and p24 are, on average, highly conserved: the consensus amino acid was observed in a super-majority (≥80%) of the sequence data at 84% of positions for p17 and 94% of positions for p24. We thus expect that a binary representation of the data will be sufficient to capture useful information about correlated mutations in these proteins.

The binarized MSA data consists of B sequences $\underline{s}^{(k)}$, $k = 1, \ldots, B$, with $B = 4389$ for p17 and $B = 4003$ for p24. We write each sequence $\underline{s}^{(k)} = \{s_1^{(k)}, s_2^{(k)}, \ldots, s_N^{(k)}\}$, where N is the total length of the protein ($N = 132$ for p17 and $N = 231$ for p24), and $s_i^{(k)} \in \{0, 1\}$ is a binary variable which specifies whether there is a zero or a one at position i in sequence k. The one- and two-point correlations we obtain from the data are then

$$p_i^* = \frac{1}{B}\sum_{k=1}^{B} s_i^{(k)}, \qquad p_{ij}^* = \frac{1}{B}\sum_{k=1}^{B} s_i^{(k)} s_j^{(k)}\,. \tag{1}$$

The one-point correlations p_i^* measure the frequency of mutations at each position i, and the two-point correlations p_{ij}^* measure the frequency of pairs of mutations occuring simultaneously at two positions i, j.

1.2 Maximum entropy model for viral fitness

A suitable model for viral fitness should be able to capture the pattern of correlated mutations observed in the MSA. It has been argued for statistical inference that, in the absence of information which would lead us to select a particular model, the maximum entropy model consistent with the data should be favored because it is the least constrained model which is capable of describing the system (Jaynes, 1982).

The maximum entropy probabilistic model capable of reproducing the correlations (Eqn. 1) is the Ising model. In this framework, the probability of observing a particular sequence $\underline{s}$ is given by

$$P(\underline{s}) = \frac{e^{-E(\underline{s})}}{Z}\,, \tag{2}$$

where $E(\underline{s})$ is the energy of $\underline{s}$,

$$E(\underline{s}) = -\sum_{i=1}^{N} h_i\, s_i - \sum_{i=1}^{N-1} \sum_{j=i+1}^{N} J_{ij}\, s_i\, s_j\,, \tag{3}$$

and Z, the partition function, is a normalizing constant which ensures that the probability of observing all possible sequences adds up to one,

$$Z = \sum_{\underline{s}} e^{-E(\underline{s})}\,. \tag{4}$$

Here the sum over $\underline{s}$ represents a sum over all the 2^N possible binary sequences of length N. Following the conventions of statistical physics, we refer to the N parameters $\{h_i\}$ in (Eqn. 3) as fields, and the $N(N-1)/2$ parameters $\{J_{ij}\}$ as couplings.

Note that the probability measure (Eqn. 2) places greater weight on sequences with *low* energies. Thus, the lower the energy of a given sequence, the *higher* its predicted fitness.

1.3 The inverse Ising problem

In order to fit an Ising model to the MSA data, we must solve the inverse Ising problem: we must determine the fields $\{h_i\}$ and couplings $\{J_{ij}\}$ appearing in (Eqn. 3) such that the one- and two-point correlations obtained from the Ising model

$$p_i = \langle s_i \rangle \equiv \sum_{\underline{s}} s_i\, \frac{e^{-E(\underline{s})}}{Z}, \qquad p_{ij} = \langle s_i\, s_j \rangle \equiv \sum_{\underline{s}} s_i\, s_j\, \frac{e^{-E(\underline{s})}}{Z}\,, \tag{5}$$

match the empirical correlations from the MSA (Eqn. 1). We use $\langle\cdot\rangle$ in (Eqn. 5) to denote an average with respect to the probability measure (Eqn. 2). Note that this average depends upon the value of the $\{h_i\}$ and $\{J_{ij}\}$.

Maximum likelihood estimation, a fundamental method of statistical inference, provides one method of solution of the inverse problem. The approach is as follows. We can compute via (Eqn. 2) the probability or *likelihood* of observing the collection of MSA data $\{\underline{s}^{(k)}\}$ as a function of the parameters $\{h_i\}$, $\{J_{ij}\}$:

$$\ell(\{\underline{s}^{(k)}\}|\{h_i\},\{J_{ij}\}) = \prod_{k=1}^{B} \frac{e^{-E(\underline{s}^{(k)})}}{Z}\,. \tag{6}$$

For convenience, we will use the logarithm of the likelihood, divided by the number of sequences B,

$$\hat{\ell}(\{\underline{s}^{(k)}\}|\{h_i\},\{J_{ij}\}) = -\log Z + \sum_i p_i^* h_i + \sum_{i=1}^{N-1} \sum_{j=i+1}^{N} p_{ij}^* J_{ij}. \tag{7}$$

The fields and couplings $\{h_i\}$, $\{J_{ij}\}$ which maximize the likelihood (or equivalently, the average log-likelihood (Eqn. 7)) then satisfy

$$\frac{\partial \hat{\ell}}{\partial h_i} = \langle s_i \rangle - p_i^* = 0, \qquad \frac{\partial \hat{\ell}}{\partial J_{ij}} = \langle s_i\, s_j \rangle - p_{ij}^* = 0. \tag{8}$$

Thus we see that the $\{h_i\} = \{h_i^*\}$, $\{J_{ij}\} = \{J_{ij}^*\}$ which maximize the likelihood of the experimental data, (if they exist and are finite, see (Barton and Cocco, 2013)), also reproduce the experimentally measured one- and two-point correlations. While maximization of (Eqn. 7) provides a method by which the desired $\{h_i^*\}, \{J_{ij}^*\}$ may be obtained, this equation has no analytical solution for $N > 3$, and, because the number of terms in the partition function scales exponentially with the system size, direct numerical maximization is precluded for systems with $N \gtrsim 20$.

For practical purposes, to control the effects of undersampling and noise in the data, we also add a *regularization* term

$$-\gamma \sum_{i=1}^{N-1} \sum_{j=i+1}^{N} J_{ij}^2 \tag{9}$$

to (Eqn. 7) which penalizes large couplings in the inference. Here γ is a variable that controls the strength of the regularization. The addition of this term to (Eqn. 7) can be interpreted in a Bayesian sense as assuming a Gaussian prior distribution for the couplings $\{J_{ij}\}$. The regularization introduces a compromise between *structure*, in the form of couplings between sites, and *predictive power*, the agreement between the inferred correlations and those obtained from data. Regularization is also helpful from a numerical perspective as it accelerates the fitting of model parameters.

1.4 Solution of the inverse Ising problem

To solve the inverse Ising problem, we used the method of selective cluster expansion (SCE) (Cocco and Monasson, 2011, 2012; Barton and Cocco, 2013), which constructs an estimate for the $\{h_i^*\}$, $\{J_{ij}^*\}$ by directly solving (Eqn. 7), including the regularization term (Eqn. 9), for small subsets of the full system and combining the results. For thorough reviews of this method, see (Cocco and Monasson, 2012; Barton and Cocco, 2013).

In some cases, the SCE algorithm became too computationally expensive (when the number or size of clusters in the expansion becomes too large) to continue running effectively and was stopped before convergence. To find acceptable values for the $\{h_i\}, \{J_{ij}\}$ we then employed a simple gradient descent learning algorithm (Nocedal and Wright, 1999), using the couplings and fields obtained from the SCE as a starting point. The learning algorithm consists of iterative rounds of Monte Carlo simulation of the Ising model to determine the one- and two-point correlations, followed by an update to the fields and couplings

$$h_i \to h_i + \alpha(p_i - p_i^*), \qquad J_{ij} \to J_{ij} + \alpha(p_{ij} - p_{ij}^* + 2\,\gamma\, J_{ij})\,. \tag{10}$$

The multiplier α was chosen adaptively at each step in the algorithm. The γ-dependent term in (Eqn. 10) for the couplings $\{J_{ij}\}$ enforces the regularization in the same way as (Eqn. 9).

The quality of the fit of the inferred Ising model to data was measured by errors on the one-point correlations $\{p_i\}$ and two-point connected correlations $\{c_{ij}\} = \{p_{ij} - p_i p_j\}$ obtained from Monte Carlo simulations,

$$\epsilon_p = \left(\frac{1}{N} \sum_{i=1}^{N} \frac{(p_i - p_i^*)^2}{(\delta p_i^*)^2} \right)^{\frac{1}{2}}, \quad \epsilon_c = \left(\frac{2}{N\,(N-1)} \sum_{i=1}^{N-1} \sum_{j=i+1}^{N} \frac{(c_{ij} - c_{ij}^*)^2}{(\delta c_{ij}^*)^2} \right)^{\frac{1}{2}}. \tag{11}$$

The denominators in (Eqn. 11) measure the typical fluctuations of the data expected due to finite sampling (Cocco and Monasson, 2011),

$$\begin{aligned}
\delta p_i^* &= \sqrt{\frac{p_i^*(1-p_i^*)}{B}}, \\
\delta p_{ij}^* &= \sqrt{\frac{p_{ij}^*(1-p_{ij}^*)}{B}}, \\
\delta c_{ij}^* &\approx \delta p_{ij}^* + p_i^* \delta p_j^* + p_j^* \delta p_i^* \,.
\end{aligned} \tag{12}$$

The inferred Ising model is considered to fit the data well when $\epsilon_p, \epsilon_c \lessapprox 1$. This condition implies that the correlations of the inferred Ising model match those in the MSA to within the uncertainty of the MSA correlations due to finite sample size. If while running the SCE algorithm or the learning algorithm a set of $\{h_i\}, \{J_{ij}\}$ satisfying $\epsilon_p, \epsilon_c \lessapprox 1$ was found, the fields and couplings were recorded and the algorithm terminated.

1.5 Model selection

For both p17 and p24 we tested 100 different values of the regularization strength, chosen over two orders of magnitude in equally-spaced logarithmic steps around an initial guess of

$$\gamma = \frac{1}{10\,B\,\bar{p}^*(1-\bar{p}^*)}\,, \qquad \bar{p}^* = \frac{1}{N}\sum_{i=1}^{N} p_i^*\,, \tag{13}$$

which was shown to give good results in similar problems (Cocco and Monasson, 2011). By construction, the inferred Ising models obtained using different values of the regularization strength typically fit the one- and two-point correlations $\{p_i^*\}$, $\{p_{ij}^*\}$ quite well (otherwise it would not be possible to achieve $\epsilon_p, \epsilon_c \lessapprox 1$).

To choose between many Ising models inferred with different values of the regularization strength γ, all of which fit the one- and two-point correlations well, we made a comparison with higher order statistics. Unlike the $\{p_i\}$ and $\{p_{ij}\}$, higher order statistics, such as three-point correlations or the probability $P(n)$ of observing sequences with n mutations with respect to the wild-type sequence, are not constrained in the inference problem. A good fit to these higher order statistics is thus a measure of the *predictive power* of the inferred Ising model. Because mutations in the p17 and p24 proteins are rare at most sites ($\bar{p}^* = 0.09$ for p17 and $\bar{p}^* = 0.03$ for p24), typical three-point correlations are small, and thus more sensitive to noise due to finite sampling. To choose a "best" model for making fitness predictions, we selected the inferred Ising model which obtained the best fit to the $P(n)$ mutations curve, which is less sensitive undersampling issues.

2. Numerical inference of multiclade Potts viral fitness landscapes

2.1 HIV-1 clade B p17 and p24 sequence data for model parameterization

Multiple sequence alignments of the HIV-1 p17 and p24 proteins comprising 2474 and 2136 DNA sequences, respectively, were downloaded from the Los Alamos National Laboratory HIV database (http://www.hiv.lanl.gov). Sequences were restricted to HIV-1 subtype B, and limited to those derived from drug-naïve hosts and not classified in the database as "problematic." The sequence alignments were processed to remove insertions with respect to the HXB2 reference sequence (Leitner, 2005), and translated into the cognate protein sequence. Ambiguous codons containing unknown bases or gaps were translated as an unknown amino acid residue. The one and two-position amino acid residue frequencies, $\mathrm{P1}_i$ and $\mathrm{P2}_{ij}$, were computed from the multiple sequence alignments (MSA) as the fitting targets for inference of the Potts model parameters, h_i and J_{ij}.

2.2 Gauge fixing of Potts model parameters

Following Morcos *et al.*, dependencies between the one and two-position marginals allow us to define an arbitrary reference state for each h_i vector and J_{ij} matrix with no loss of generality by setting $h_i(A_i = a) = 0$ and $J_{ij}(A_i = a, A_j) = J_{ij}(A_i, A_j = a) = 0$, where a is a particular amino acid residue (Morcos, 2012). Mathematically, the Potts Hamiltonian (Eqn. 14) possesses a "gauge invariance," wherein the h_i and J_{ij} parameter values may be modified in a coordinated fashion to leave the value of E unchanged (Weigt, 2009). Elimination of this degeneracy in the model is achieved by specifying reference energies, and is known as "gauge fixing." We observe that this model degeneracy and gauge fixing necessitates that one must be circumspect in assigning physical interpretations to individual model parameters.

In this work, we elect to pin to zero the h_i and J_{ij} elements corresponding to the most probable amino acid in each position. Fitting of the remaining vector and matrix elements to reproduce the observed one and two-position amino acid frequencies is a convex inverse inference problem possessing a unique solution corresponding to the maximum likelihood estimates of the Potts model parameters (Weigt, 2009; Morcos, 2012; Ferguson, 2013).

2.3 Model simplification

The number of parameters in the Potts Hamiltonian described in (Eqn. 14) is $mq + m(m-1)q^2/2$, where m is the number of positions in the protein, and q=21 is the number of natural amino acid residues, plus the unknown residue, that may occupy each position. To simplify the model, and accelerate and stabilize numerical fitting of its parameters, we reduced the number of model parameters by truncating the h_i vector at each position and J_{ij} matrix at each pair of positions to contain only those residues that are actually observed in each position within the MSA. This simplification restricts the generality of the inferred model, rendering it unable to assign energies to viral strains containing residues in particular positions that were not observed within the training data. For sufficiently many sequences in the MSA, we anticipate that viable mutant strains containing point mutations not present within this data will be rare.

In principal, this model simplification is not required, and it is possible to fit a model containing parameters for all amino acid residues at all positions. This would require additional model regularization to stabilize numerical fitting of the model by adding pseudo-counts to the $\mathrm{P1}_i$ marginals in an analogous manner as described below for the $\mathrm{P2}_{ij}$ marginals, and would significantly increase the computational effort required for numerical parameterization.

2.4 Bayesian regularization 1: Addition of pseudo-counts to $\mathrm{P2}_{ij}$ *marginals*

The viral sequences constituting the MSA represent an incomplete sampling of the mutational space available to the HIV-1 virus. Correspondingly, it is expected that not all possible pairs of amino acid mutations will be present within the data. (In contrast, the model is restricted as described above, such that only those point mutations that are observed within the data are represented in the model.)

To reflect this incomplete knowledge, we wish to assert our belief that these pairs of mutations are not infinitely improbable, but just sufficiently unlikely that they are not represented within our finite sequence data. We encode this assertion as a Bayesian prior by adding pseudo-counts to our two-position frequency computations (Sivia and Skilling, 2003). Physically, this can be interpreted as asserting that every possible pair of amino acid mutations is observed a small number of times, and adding these counts to those computed from the MSA. In practice, we add a small value, $\gamma_2/n_i n_j$, to each element of the $\mathrm{P2}_{ij}$ matrix for each pair of positions – where n_i is the number of single residue parameters contained within the truncated h_i vector (see above) – and iteratively rescale the rows and columns of the matrix to reproduce the corresponding $\mathrm{P1}_i$ and $\mathrm{P1}_j$ marginals.

2.5 Bayesian regularization 2: Gaussian prior distribution on Potts model parameters

To further stabilize numerical fitting of our model, we adopt a second form of regularization on the Potts model parameters to regularize growth of the absolute parameter values. This regularization was imposed in response to empirical observations of coupled groups of h_i and J_{ij} elements exhibiting uncontrolled absolute growth in numerical fitting of the model. We enforce this regularization in the context of a Bayesian approach to parameter estimation (see below), and adopt a Gaussian prior distribution over the Potts model parameters, $\vec{\theta} = \{h_i, J_{ij}\}$,

$$P(\vec{\theta}) = P(\{h_i\})P(\{J_{ij}\}) = \prod_i \prod_q \exp\left[-\lambda_h \left\| h_i(q) \right\|_2\right] \cdot \prod_i \prod_{j>i} \prod_q \prod_r \exp\left[-\lambda_J \left\| J_{ij}(q,r) \right\|_2\right], \tag{14}$$

Physically, this prior distribution penalizes large values of model parameters, reflecting our belief that the parameters should not grow arbitrarily large, and restraining uncontrolled growth of coupled groups of parameters. We observe that substituting the L_2-norms for L_1-norms in the above expression corresponds to the specification of a Laplacian prior distribution, which represents another common choice of regularizing prior (Sivia and Skilling, 2003).

2.6 Numerical inference of parameters by semianalytical gradient descent

In a generalization of our previous approach for Ising parameter inference (Ferguson, 2013), the parameters of the Potts model were fitted using a semianalytical extension of the iterative gradient descent implemented by Mora and Bialek (Mora and Bialek, 2011). Adopting a Bayesian perspective, we seek to maximize the likelihood, L, of the model parameters, $\vec{\theta} = \{h_i, J_{ij}\}$, given the observed protein sequences in the MSA (the "data", D) under the adopted prior distribution, $P(\vec{\theta})$. The likelihood of the model given the data is,

$$L(D|\vec{\theta}) = P(\vec{\theta}|D) \propto P(D|\vec{\theta})P(\vec{\theta}). \tag{15}$$

$P(\vec{\theta})$ is given by (Eqn. 14), and, assuming the sequences in the MSA to be independent and identically distributed,

$$P(D|\vec{\theta}) = \prod_{\{\vec{A}\}} P(\vec{A})^{P^{obs}(\vec{A})K}, \tag{16}$$

where $P\left(\vec{A}\right)$ is the probability predicted by the Potts model of sequence $\vec{A}$ within the space of all possible sequences given by (Eqn. 14), $P^{obs}\left(\vec{A}\right)$ is the observed probability of sequence $\vec{A}$ within the MSA, and K is the number of sequences in the MSA.

It may be shown that the likelihood in (Eqn. 15) is maximized with respect to the model parameters $\vec{\theta} = \left\{h_i, J_{ij}\right\}$ when,

$$\mathrm{P1}_i\left(q\right) = \mathrm{P1}_i^{obs}\left(q\right) + \tfrac{2}{K}\lambda_h h_i\left(q\right), \tag{17a}$$

$$\mathrm{P2}_{ij}\left(q,r\right) = \mathrm{P2}_{ij}^{obs}\left(q,r\right) + \tfrac{2}{K}\lambda_J J_{ij}\left(q,r\right). \tag{17b}$$

The parameter values satisfying these relations correspond to the maximum a posterior (MAP) estimates of the model parameters, and may be viewed as regularized versions of the maximum likelihood estimates (Sivia and Skilling, 2003).

In practice, the MAP estimate may be numerically computed by iteratively adjusting the Potts model parameters from some initial guess until (Eqns. 17a,b) are satisfied, and the model parameters converge. In a generalization of our previous approach (Ferguson, 2013), the Potts Hamiltonian (Eqn. 14) admits an analytical expression for the Jacobian,

$$\mathbf{J} = \frac{\partial\left\{\mathrm{P1}_i(q), \mathrm{P2}_{ij}(q,r)\right\}}{\partial\left\{h_i(q), J_{ij}(q,r)\right\}}, \tag{18}$$

enabling the formulation of a multidimensional Newton search for the MAP parameter estimates. (Numerical estimates of the gradients would require double the number of model probability evaluations to estimate gradients, resulting in a less efficient multidimensional secant search subject to greater sampling noise.)

Using the analytical expressions for the gradients, the Taylor expansions for the one and two-position amino acid probabilities predicted by the model, $\left\{\mathrm{P1}_i, \mathrm{P2}_{ij}\right\}$, around target probabilities, $\left\{\mathrm{P1}_i^*, \mathrm{P2}_{ij}^*\right\}$, retaining in each expansion only the term in each probability marginal's "own" model parameter are,

$$\mathrm{P1}_i^*\left(q\right) \quad = \mathrm{P1}_i\left(q\right) + \frac{\partial \mathrm{P1}_i\left(q\right)}{\partial h_i\left(q\right)}\Delta h_i\left(q\right) + \ldots$$

$$= \mathrm{P1}_i(q) + \mathrm{P1}_i(q)\left[\mathrm{P1}_i(q) - 1\right]\Delta h_i(q) + \ldots, \tag{19a}$$

$$\mathrm{P2}^*_{ij}(q,r) = \mathrm{P2}_{ij}(q,r) + \frac{\partial \mathrm{P2}_{ij}(q,r)}{\partial J_{ij}(q,r)}\Delta J_{ij}(q,r) + \ldots$$
$$= \mathrm{P2}_{ij}(q,r) + \mathrm{P2}_{ij}(q,r)\left[\mathrm{P2}_{ij}(q,r) - 1\right]\Delta J_{ij}(q,r) + \ldots. \tag{19b}$$

These expressions may be rearranged to establish the Newton steps,

$$\Delta h_i(q) = \gamma_h \left[\frac{\mathrm{P1}^*_i(q) - \mathrm{P1}_i(q)}{\mathrm{P1}_i(q)\left(\mathrm{P1}_i(q) - 1\right)}\right], \tag{20a}$$

$$\Delta J_{ij}(q) = \gamma_J \left[\frac{\mathrm{P2}^*_{ij}(q,r) - \mathrm{P2}_{ij}(q,r)}{\mathrm{P2}_{ij}(q,r)\left(\mathrm{P2}_{ij}(q,r) - 1\right)}\right], \tag{20b}$$

where γ_h and γ_J are softening parameters to improve numerical stability of the fitting trajectory. Under the Gaussian priors on the model parameters, the target probabilities are given by (Eqns. 17a,b), and the Newton steps become,

$$\Delta h_i(q) = \gamma_h \left[\frac{\mathrm{P1}^{obs}_i(q) - \mathrm{P1}_i(q) + \frac{2}{K}\lambda_h h_i(q)}{\mathrm{P1}_i(q)\left(\mathrm{P1}_i(q) - 1\right)}\right], \tag{21a}$$

$$\Delta J_{ij}(q) = \gamma_J \left[\frac{\mathrm{P2}^{obs}_{ij}(q,r) - \mathrm{P2}_{ij}(q,r) + \frac{2}{K}\lambda_J J_{ij}(q,r)}{\mathrm{P2}_{ij}(q,r)\left(\mathrm{P2}_{ij}(q,r) - 1\right)}\right]. \tag{21b}$$

2.7 Monte-Carlo evaluation of model probabilities

Repeated application of the Newton steps in (Eqns. 21a,b) iteratively converge the model parameters to their MAP estimates. At each iteration, the one and two-position amino acid probabilities predicted by the model, $\{\mathrm{P1}_i, \mathrm{P2}_{ij}\}$, at the current parameter values are computed by Markov-chain Monte-Carlo (MCMC) (Ferguson, 2013). We implement this process by initializing the Markov chain to a protein sequence vector, $\vec{A}$, in which each position is occupied by the most probable amino acid residue observed in the data, and the Markov chain is then evolved for a fixed number of steps, M, by proposing point mutations in the protein sequence and accepting or rejecting these mutations according to the Metropolis acceptance criterion (Frenkel and Smit, 2002). The one and two-position amino acid frequency marginals are estimated from the series

of sequences realized in the Markov chain. Evaluation of these probabilities at each Newton step is the principal computational cost of our numerical fitting approach.

2.8 Parameter initialization

To accelerate the numerical fitting of the model parameters, initial values for the h_i vector elements were specified using the $\mathrm{P1}_i^{obs}$ values by assuming an independent site model (i.e., by setting all J_{ij} elements in (Eqn. 14) to zero). All J_{ij} matrix elements were initialized as zero.

2.9 Numerical results

We inferred the MAP estimates of the Potts $\{h_i, J_{ij}\}$ parameter values for p24 by performing N =10,000 Newton steps with softening parameters $\gamma_h = \gamma_J = 0.01$, and regularization parameters γ_2 =0.1, λ_h/K =0.0, λ_J/K =2.5×10^{-3}. To further stabilize the numerical fitting, the J_{ij} values were only updated every other Newton step, and the maximum Newton step at each iteration limited to 0.01. M =8,000,000 MCMC steps were performed at each Newton step, and the $\{\mathrm{P1}_i, \mathrm{P2}_{ij}\}$ marginals evaluated by sampling every 10th realization of the MCMC trajectory.

The p17 model parameters were inferred by performing N =25,000 Newton steps. Identical fitting parameters were implemented to p24, with the following two exceptions. Since the protein is shorter, the number of MCMC steps per Newton iteration was reduced to M =2,000,000. A milder J_{ij} regularization strength of λ_J/K =1.0×10^{-3} was admitted without compromising the stability of the fitting procedure.

In the case of p24, an in-house C++ implementation of the numerical fitting algorithm parallelized over 32 2.66 GHz Intel Xeon CPU cores under MPI required approximately 11 days of wall time (~1.4 years of CPU time) to converge. For p17, 5 days of wall time using 16 cores was required (~80 days of CPU time).

In Figs. 1 and 2 we present a comparison of the p17 and p24 one and two-position amino acid frequencies in the MSA to those computed by performing MCMC sampling from the final Potts model.

3. Comparison with experimental measurements of replicative capacity

3.1 Correlation with energy for the uncorrected, regularized Ising model

Once we chose the best fitting Ising model for p17 and p24, we computed the energy (Eqn. 3) of the NL4-3 sequence, $E(\underline{s}_{\text{NL4}-3})$, as well as the energy of each mutant which was tested in the replicative capacity (RC) experiments. Assuming that the RC of a virus with a given sequence is proportional to its probability (Eqn. 2) in the inferred Ising model, we have

$$\frac{\text{RC}_{\text{mutant}}}{\text{RC}_{\text{NL4}-3}} = \frac{e^{-E(\underline{s}_{\text{mutant}})}}{e^{-E(\underline{s}_{\text{NL4}-3})}}, \tag{22}$$

so that

$$\Delta E = E(\underline{s}_{\text{mutant}}) - E(\underline{s}_{\text{NL4}-3}) = \log\left(\frac{\text{RC}_{\text{mutant}}}{\text{RC}_{\text{NL4}-3}}\right). \tag{23}$$

Thus, we expect the difference in energy between the wild-type and a mutant sequence should give an indication of (the logarithm of) the ratio of their RC. For comparison with previous results, we show the differences in energy computed for the original, unregularized Ising model (Ferguson *et al.*, 2013) in Figure 3 and Table 1.

Table 2 shows the difference in energy ΔE between the NL4-3 sequence and each mutant, as well as the RC ratio $\text{RC}_{\text{mutant}}/\text{RC}_{\text{NL4}-3}$, with $\text{RC}_{\text{NL4}-3} = 1$ for convenience. The correlation between ΔE and the RC ratio is strong and highly significant (Pearson $\rho = -0.82$, $p = 1.89 \times 10^{-11}$).

In practice, we may be more interested in more coarse-grained measures of viral fitness: will a virus with a given sequence be able to replicate with an efficiency similar to the wild-type, or will it be significantly impaired? To explore this point, we grouped the experimentally tested mutants into two categories, *fit* (RC ratio > 0.5) and *unfit* (RC ratio ≤ 0.5). We then fit a linear classifier to the data using logistic regression (Hastie *et al.*, 2009), with ΔE as the predictor variable and the fitness class, *fit* or *unfit*, as the outcome. In this model the probability that a mutant with $\Delta E = x$ is assigned to the *fit* category is given by

$$P(fit) = \frac{e^{\beta_0+\beta x}}{1+e^{\beta_0+\beta x}}, \qquad P(unfit) = 1 - P(fit) = \frac{1}{1+e^{\beta_0+\beta x}}, \tag{24}$$

with β_0 and β determined by maximum likelihood. Analysis was performed with the statistical package R (R Core Team, 2012). As a measure of classifier performance, we also report the area under the receiver operating characteristic curve (AUROC), which is formed by plotting the true positive rate of the classifier versus as a function of the false positive rate for all potential values of the threshold (Peterson *et al.*, 1954). An AUROC value of 1 represents perfect classification, while 0.5 represents classification accuracy equivalent to chance.

In this case we found the maximum likelihood logistic function (Eqn. 24) with $\beta_0 = 7.346$ and $\beta = -0.991$. The inferred crossover point is $\Delta E_{\text{cross}} = 7.413$; mutants with $\Delta E < \Delta E_{\text{cross}}$ are assigned to the *fit* group, while those with $\Delta E > \Delta E_{\text{cross}}$ are predicted to be *unfit*. This

classifier is quite accurate, classifying 38 out of the 43 mutants (88.4%) correctly (AUROC= 0.88). The accuracy and significance of this classifier are also stable under reasonable changes of the classification criterion (e.g. RC ratio cutoff of 0.4 instead of 0.5).

Excluding p17 mutants, we find for p24 a slightly stronger correlation between the energy and fitness (Pearson $\rho = -0.84$, $p = 4.78 \times 10^{-11}$). The maximum likelihood classifier trained on this data has $\beta_0 = 7.322$ and $\beta = -1.019$. This gives a cutoff of $\Delta\tilde{E}_{\mathrm{cross}} = 7.185$, which classifies 35 of 38 mutants (92.1%) correctly (AUROC= 0.91).

Mutations	Gag protein	ΔE	$\frac{\mathrm{RC_{mutant}}}{\mathrm{RC_{NL4-3}}}$
186I	p24	75.76	0.67
269E	p24	40.45	0
186I269E	p24	Infinity	0
295E	p24	19.83	0.94
186I295E	p24	Infinity	0
181R	p24	41.64	0
310T	p24	3.27	0.9
181R310T	p24	Infinity	0
182S	p24	22.15	0.97
198V	p24	Infinity	0
182S198V	p24	Infinity	0
179G	p24	53.11	0.8
229K	p24	41.64	0.75
179G229K	p24	94.03	0
174G	p24	Infinity	0
243P	p24	63.67	0.36
174G243P	p24	Infinity	0
168I	p24	35.60	0.82
315G	p24	16.13	0.86
168I315G	p24	Infinity	0.36
331R	p24	8.78	0.96
186I331R	p24	Infinity	0
302R	p24	8.12	0.85
302R315G	p24	Infinity	0.45
315G331R	p24	Infinity	0.86
190I	p24	38.54	0.39
190I302R	p24	Infinity	0.56
219Q	p24	3.75	0.99
242N	p24	5.70	0.91
219Q242N	p24	7.82	0.99
146P	p24	4.23	0.99
147L	p24	0.44	0.95
146P147L	p24	3.59	1
326S	p24	1.61	0.64
310T326S	p24	7.55	1.04
173T	p24	2.93	0.88
173T286K	p24	1.91	0.94
173T286K147L	p24	1.14	1
12K	p17	0.31	0.95
12K54A	p17	1.41	1.01
86F	p17	4.57	1.05
92M	p17	5.31	0.99
86F92M	p17	Infinity	1.07

Table 1: Table of energy differences ΔE computed by the unregularized Ising model (Ferguson *et al.*, 2013) and replicative capacity ratios $\mathrm{RC_{mutant}}/\mathrm{RC_{NL4-3}}$ for the experimentally tested mutants. All mutations are defined with respect to the NL4-3 sequence. Labeling of mutation positions is with respect to the start of Gag. The energy of the NL4-3 sequence is 3.43 for p17 and 2.98 for p24.

Mutations	Gag protein	ΔE	$\frac{\mathrm{RC}_{\mathrm{mutant}}}{\mathrm{RC}_{\mathrm{NL4-3}}}$
186I	p24	6.16	0.67
269E	p24	7.33	0
186I269E	p24	13.50	0
295E	p24	5.32	0.94
186I295E	p24	11.48	0
181R	p24	8.46	0
310T	p24	2.19	0.90
181R310T	p24	10.65	0
182S	p24	3.43	0.97
198V	p24	7.87	0
182S198V	p24	11.30	0
179G	p24	7.10	0.80
229K	p24	6.63	0.75
179G229K	p24	13.72	0.00
174G	p24	9.10	0
243P	p24	7.08	0.36
243P174G	p24	16.18	0.00
168I	p24	6.00	0.82
315G	p24	3.18	0.86
168I315G	p24	10.59	0.36
331R	p24	3.69	0.96
186I331R	p24	9.85	0.00
302R	p24	4.06	0.85
302R315G	p24	8.63	0.45
315G331R	p24	6.87	0.86
190I	p24	4.51	0.39
190I302R	p24	8.57	0.56
219Q	p24	1.98	0.99
242N	p24	3.02	0.91
219Q242N	p24	4.37	0.99
146P	p24	1.94	0.99
147L	p24	0.58	0.95
146P147L	p24	2.10	1.00
326S	p24	1.11	0.64
310T326S	p24	4.05	1.04
173T	p24	2.14	0.88
173T286K	p24	2.89	0.94
173T286K147L	p24	2.26	1.00
12K	p17	0.27	0.95
12K54A	p17	1.55	1.01
86F	p17	2.89	1.05
86F92M	p17	7.83	1.07
92M	p17	4.35	0.99

Table 2: Table of regularized Ising model energy differences ΔE (Eqn. 23) and replicative capacity ratios $\mathrm{RC}_{\mathrm{mutant}}/\mathrm{RC}_{\mathrm{NL4-3}}$ for the experimentally tested mutants. All mutations are defined with respect to the NL4-3 sequence. Labeling of mutation positions is with respect to the start of Gag. The energy of the NL4-3 sequence is 1.675 for p17 and 3.736 for p24.

3.2 Correction for the effects of mutations

The inverse Ising procedure infers an Ising model approximates the empirical distribution obtained from the MSA data, consistent with the one- and two-point correlations. Predicted energies/fitnesses then provide estimates for the likelihood of measuring a given sequence. However, given that viruses can mutate during replication, the prevalence of a particular strain in the population does not necessarily correspond with its ability to replicate. For instance, viruses which are completely unable to replicate will still occasionally be produced due to mutation of similar, replication-competent viruses. The following is a very simple attempt to account for these effects.

We assume that the steady state distribution of viruses with sequence $\underline{s}$ is given by the Ising model probability measure

$$P(\underline{s}) = \frac{e^{-E(\underline{s})}}{Z}. \tag{25}$$

Let us consider a simple model where replication takes place in discrete steps, and after each step the old population is replaced by the new. For all viruses independent of sequence, the probability of mutating during a replication step is $\alpha \ll 1$. When a mutation occurs, one site is chosen at random and its value is flipped from a zero to a one, or vice versa.

Let us call $Q(\underline{s})$ the fraction of all the viruses produced in each replication step which are the offspring of viruses with sequence $\underline{s}$, including those which mutate during replication. Then the steady state condition gives

$$P(\underline{s}) = (1-\alpha)Q(\underline{s}) + \frac{\alpha}{N} \sum_{\underline{s}' | d_{\underline{s},\underline{s}'}=1} Q(\underline{s}'), \tag{26}$$

where $d_{\underline{s},\underline{s}'}$ is the Hamming distance between sequences $\underline{s}$ and $\underline{s}'$, such that the sum in (Eqn. 26) is a sum over all sequences $\underline{s}'$ separated from sequence $\underline{s}$ by a single mutation.

We will now solve for $Q(\underline{s})/P(\underline{s})$ to first order in α. This ratio represents the number of expected offspring that each virus with sequence $\underline{s}$ will produce in each replication step. Rearranging (Eqn. 26) and dividing through by $P(\underline{s})$ we have

$$\frac{Q(\underline{s})}{P(\underline{s})} = \frac{1}{1-\alpha}\left(1 - \frac{\alpha}{N} \sum_{\underline{s}' | d_{\underline{s},\underline{s}'}=1} \frac{Q(\underline{s}')}{P(\underline{s})}\right). \tag{27}$$

To zeroth order in α, $Q(\underline{s})$ is simply equal to $P(\underline{s})$, so (Eqn. 27) is

$$\frac{Q(\underline{s})}{P(\underline{s})} = 1 + \alpha\left(1 - \frac{1}{N} \sum_{\underline{s}' | d_{\underline{s},\underline{s}'}=1} \frac{P(\underline{s}')}{P(\underline{s})}\right) + \mathcal{O}(\alpha^2). \tag{28}$$

The interpretation of (Eqn. 28) is physically intuitive. If the sequences which differ from $\underline{s}$ by a single mutation occupy on average a *larger* fraction of the population than $P(\underline{s})$, then in the dynamics there is a net flow *in* to sequence $\underline{s}$ from its (mutational) neighbors, thus

under the steady state assumption $Q(\underline{s})$ must be smaller than $P(\underline{s})$. Conversely, if the fitness of neighbors of $\underline{s}$ is *smaller* than $P(\underline{s})$, the net flow due to mutation is *out*, and $Q(\underline{s}) > P(\underline{s})$.

We can also use (Eqn. 28) to compute an energy $\tilde{E}(\underline{s})$, which differs from $E(\underline{s})$ by terms of order α, which approximates the distribution for the number of offspring produced by each sequence in each replication step, rather than the total population fraction. Up to a constant which is independent of the sequence $\underline{s}$,

$$\tilde{E}(\underline{s}) = E(\underline{s}) - \alpha \left(1 - \frac{1}{N} \sum_{\underline{s}' | d_{\underline{s},\underline{s}'}=1} \exp\left[E(\underline{s}) - E(\underline{s}')\right] \right) + \mathcal{O}(\alpha^2)\,. \tag{29}$$

An analogous equation can be similarly derived for Potts models. In this case the sum over sequences $\underline{s}'$ a Hamming distance of 1 away from $\underline{s}$ also includes a sum over different possible amino acids at each site, so that the normalizing factor is not $1/N$ but $1/Nq$, where q is the number of states.

Adding in this correction to the energy improves the correpsondence with real fitness measurements. For most mutants the correction is very small; however, preferentially for the mutants which are unable to replicate, the corrections are much larger, leading to better separation between mutants with zero and finite replicative capacities. For the following computations we used $\alpha = 3\,N \times 10^{-4}$, though the results do not depend strongly on the specific value of α.

Table 3 shows the difference in the corrected energy $\Delta\tilde{E}$ for the regularized Ising model between the NL4-3 sequence and each mutant and the RC ratio $\mathrm{RC}_{\mathrm{mutant}}/\mathrm{RC}_{\mathrm{NL4-3}}$. This data is also reproduced in Figure 4. The correlation between $\Delta\tilde{E}$ and the RC ratio is improved relative to the uncorrected energy (Pearson $\rho = -0.83$, $p = 3.73 \times 10^{-12}$).

As before, we can train a linear classifier on the data with mutants categorized into *fit* (RC ratio > 0.5) and *unfit* (RC ratio ≤ 0.5) classes. Here the maximum likelihood logistic function (Eqn. 24) is obtained with $\beta_0 = 7.451$ and $\beta = -0.960$. The inferred crossover point is $\Delta\tilde{E}_{\mathrm{cross}} = 7.761$; mutants with $\Delta\tilde{E} < \Delta\tilde{E}_{\mathrm{cross}}$ are assigned to the *fit* group, while those with $\Delta\tilde{E} > \Delta\tilde{E}_{\mathrm{cross}}$ are predicted to be *unfit*. In this case 39 of the 43 mutants (90.7%) are classified correctly (AUROC= 0.93), and the difference between the classes is highly significant (Mann-Whitney $U = 32$, $p = 4.51 \times 10^{-7}$).

If we focus on p24 alone we find a slightly stronger correlation between the energy and fitness (Pearson $\rho = -0.85$, $p = 1.42 \times 10^{-11}$). The maximum likelihood classifier trained on this data has $\beta_0 = 7.298$ and $\beta = -0.968$. This gives a cutoff of $\Delta\tilde{E}_{\mathrm{cross}} = 7.539$, which classifies 35 of 38 mutants (92.1%) correctly (AUROC= 0.91), with a stronger difference between classes (Mann-Whitney $U = 4$, $p = 2.38 \times 10^{-9}$). Figure 5 shows fitness of each mutant and whether its energy is above or below the classifier cutoff.

In contrast to the results found for the Ising model, the correction (Eqn. 29) for the Potts model is quite small. In this case the correlation between energy and RC is only slightly affected, and the accuracy of the Potts model classifier is unchanged. Figure 6 demonstrates the small perturbation of the Potts model energies due to the correction.

Mutations	Gag protein	ΔE	$\frac{\text{RC}_{\text{mutant}}}{\text{RC}_{\text{NL4-3}}}$
186I	p24	6.31	0.67
269E	p24	7.79	0.00
186I269E	p24	14.10	0.00
295E	p24	5.38	0.94
186I295E	p24	11.68	0.00
181R	p24	9.87	0.00
310T	p24	2.19	0.90
181R310T	p24	12.07	0.00
182S	p24	3.44	0.97
198V	p24	8.65	0.00
182S198V	p24	12.09	0.00
179G	p24	7.46	0.80
229K	p24	6.85	0.75
179G229K	p24	14.32	0.00
174G	p24	11.80	0.00
243P	p24	7.43	0.36
174G243P	p24	19.23	0.00
168I	p24	6.13	0.82
315G	p24	3.18	0.86
168I315G	p24	11.11	0.36
331R	p24	3.70	0.96
186I331R	p24	10.01	0.00
302R	p24	4.08	0.85
302R315G	p24	8.72	0.45
315G331R	p24	6.88	0.86
190I	p24	4.53	0.39
190I302R	p24	8.61	0.56
219Q	p24	1.98	0.99
242N	p24	3.03	0.91
219Q242N	p24	4.37	0.99
146P	p24	1.95	0.99
147L	p24	0.58	0.95
146P147L	p24	2.10	1.00
326S	p24	1.11	0.64
310T326S	p24	4.05	1.04
173T	p24	2.14	0.88
173T286K	p24	2.89	0.94
173T286K147L	p24	2.26	1.00
12K	p17	0.27	0.95
12K/54A	p17	1.55	1.01
86F	p17	2.89	1.05
86F/92M	p17	7.88	1.07
92M	p17	4.37	0.99

Table 3: Table of mutation-corrected energy differences $\Delta\tilde{E}$ (Eqn. 29) and replicative capacity ratios $\text{RC}_{\text{mutant}}/\text{RC}_{\text{NL4-3}}$ for the experimentally tested mutants. All mutations are defined with respect to the NL4-3 sequence. Labeling of mutation positions is with respect to the start of Gag. The mutation-corrected energy of the NL4-3 sequence is 1.640 for p17 and 3.672 for p24.

3.3 Classifier with Potts model energies

As presented in the sections above, we can also use energy values from the Potts model fit for training a classifier (see Table 4 for the difference in the Potts model energy between each mutant and the NL4-3). Mutants are again divided into two classes, *fit* (RC ratio > 0.5) and *unfit* (RC ratio ≤ 0.5), and the probability of a mutant being assigned to either class is taken to be a logistic function (Eqn. 24), with parameters chosen to maximize the likelihood of the data.

Focusing on p24 only, we find that the maximum likelihood is attained with the parameters $\beta_0 = 5.075$ and $\beta = -0.573$. This yields a cutoff energy of $\Delta E_{\text{cross}} = 8.850$, which correctly classifies 29 of 36 mutants (80.6%, overall AUROC= 0.82), and the difference between the classes is strong (Mann-Whitney $U = 34$, $p = 2.93 \times 10^{-4}$). The performance of this classifier is shown in Figure 7. If p17 mutants are also included, the maximum likelihood classifier has $\beta_0 = 5.075$ and $\beta = -0.573$, thus the crossover point is $\Delta E_{\text{cross}} = 8.850$. In this case 33 out of 41 mutants (80.5%) are classified correctly (AUROC= 0.82), and the difference between the classes remains significant (Mann-Whitney $U = 70$, $p = 3.98 \times 10^{-3}$). In each case here, and for the classifiers trained using Ising model energy values, leave-one-out cross-validation confirms that the expected prediction error is similar to the error rates obtained for the full set of data.

Mutations	Gag protein	ΔE	$\frac{\text{RC}_{\text{mutant}}}{\text{RC}_{\text{NL4-3}}}$
186I	p24	6.80	0.67
269E	p24	7.75	0
186I269E	p24	14.54	0
295E	p24	6.60	0.94
186I295E	p24	13.36	0
181R	p24	7.69	0
310T	p24	2.77	0.90
181R310T	p24	10.44	0
182S	p24	5.24	0.97
198V	p24	*	0
182S198V	p24	*	0
179G	p24	7.14	0.80
229K	p24	7.24	0.75
179G229K	p24	14.38	0.00
174G	p24	7.27	0
243P	p24	6.65	0.36
243P174G	p24	13.89	0.00
168I	p24	5.87	0.82
315G	p24	6.20	0.86
168I315G	p24	11.96	0.36
331R	p24	4.73	0.96
186I331R	p24	11.42	0.00
302R	p24	4.79	0.85
302R315G	p24	10.86	0.45
315G331R	p24	10.79	0.86
190I	p24	6.97	0.39
190I302R	p24	11.69	0.56
219Q	p24	2.47	0.99
242N	p24	3.61	0.91
219Q242N	p24	5.64	0.99
146P	p24	1.83	0.99
147L	p24	0.31	0.95
146P147L	p24	2.11	1.00
326S	p24	1.26	0.64
310T326S	p24	4.37	1.04
173T	p24	2.59	0.88
173T286K	p24	3.32	0.94
173T286K147L	p24	2.34	1.00
12K	p17	1.57	0.95
12K54A	p17	2.82	1.01
86F	p17	3.19	1.05
86F92M	p17	9.76	1.07
92M	p17	6.62	0.99

Table 4: Table of regularized Potts model energy differences ΔE and replicative capacity ratios $\text{RC}_{\text{mutant}}/\text{RC}_{\text{NL4-3}}$ for the experimentally tested mutants. All mutations are defined with respect to the NL4-3 sequence. Labeling of mutation positions is with respect to the start of Gag. The energy of the NL4-3 sequence is 2.81 for p17 and 4.43 for p24. (* denotes sequences with mutations not observed in the MSA, so their energies cannot be computed in the Potts model as described here.)

3.4 Comparison between Potts and Ising model descriptions of a simple toy model

In this study, we tested the fitness of sequences with mutations from the NL4-3 reference to the most commonly observed mutant amino acid at each codon (or set of codons) selected. This strategy allows for the best comparison of the tested mutants' fitness with Ising model predictions, and is a reasonable approach for a conserved protein such as Gag, where the diversity of different amino acids observed at each site is typically quite low. However, this approach may conceal some potential advantages of a Potts model over an Ising model for making fitness predictions.

Here we demonstrate, using a simple toy model, that the binary approximation (i.e. Ising model representation) of a sequence with multiple possible amino acids at each site displays several biases which can lead to inaccurate predictions. In particular, we show the following results for the Ising model.

1. The energy of mutants at a site where several different mutations are similarly likely is underestimated (equivalently, the fitness is overestimated).
2. The energy of rare mutants is severely underestimated (equivalently, the fitness is overestimated).
3. Inferred interactions tend to reflect interactions between the most common mutants.

Consider a model protein where each site is one of three possible amino acids: one "wild-type" amino acid, denoted by 0, and two mutants, denoted as 1 and 2 in order of their frequency. Each site interacts with its nearest neighboring sites. We will assume the interactions are such that the most common mutant amino acids are more likely to occur together at a neighboring pair of sites than would be expected if they were independent (i.e. there is a positive or compensatory interaction between them), while the less common amino acid mutants are *less* likely to occur together than if they were independent (i.e. there is a negative or deleterious interaction). For definiteness we will consider a system of $N = 5$ sites, with true fields $h_i(1) = -1$, $h_i(2) = -i$ with $i = \{1, 2, 3, 4, 5\}$, and true interactions $J_{i,i+1}(1, 1) = 0.5$, $J_{i,i+1}(2, 2) = -1$, and all other couplings equal to zero, reflecting the underlying fitness of each sequence. These interactions yield average mutation frequencies of the same order as those observed at variable sites in HIV protein sequences. In this toy model, the frequency of the most common mutant amino acid is similar across all sites. The frequency of the rare mutant amino acid decreases as the site index increases; at site 1, it is similar in frequency to the common mutant, while at site 5 the rare mutant is approximately 100 times less likely to be observed than the common mutant.

To assess the ability of the Potts and Ising models to describe this system, we generated a sample set of sequences according to the true model through Monte Carlo simulation and used the resulting correlations to infer Potts and Ising models which reproduce the data, as described in the sections above. We then compared the true energy to the energy computed from the inferred Potts and Ising models for sequences bearing one or two mutations. The energy of the single mutants to the most common mutant amino acid 1 and to the rare variant 2 are shown in Figures 8 and 9, respectively. Clearly we see that the Potts model is able to

accurately recover the single site mutant energies, while the Ising model underestimates the energy of common mutants (when the rarer variant is also similar in frequency) and severely underestimates the energy of the rarer mutants (in all cases), as stated in points 1) and 2) above.

We can also examine the difference in energy between double mutants and single mutants at neighboring sites to test the ability of each model to accurately recover interactions between different mutants. The difference between the double mutant and single mutant energies at neighboring sites is the coupling, $J_{i,i+1}(a, b)$, where a, b are the single mutant states. The inferred energy differences for $a = b = 1$ and $a = b = 2$ for the Potts and Ising models are plotted against their true values in Figure 10. The Potts model successfully recovers all interactions, but interactions between rare variants are more difficult to determine precisely. The Ising model is unable to distinguish between common and rare mutants, but the inferred interactions are quite similar to the true interactions between the most common mutant amino acids at each site. This shows point 3), stated above, and suggests that the Ising model should be able to successfully infer interactions between common mutations. Indeed, in this study we have found that the Ising model energy does an excellent job of predicting the fitness of similar sequences with common mutations.

Note that, while the simple toy model considered here is small ($N = 5$ sites), the results are general and do not depend on the system size. Additionally, though we have used energy as the comparison variable for transparency and analytical convenience, the same general results should hold even if one compares with sequence prevalence data coming from an evolutionary dynamics (Shekhar *et al.*, 2013).

4. Statistical notes

Here we define and briefly explain the statistical tests employed in this paper. For further information, see standard references (e.g. (DeGroot and Schervish, 2002)).

Pearson's correlation measures the linear relationship between two sets of variables. If we call these sets of variables $X = \{x_1, \dots, x_n\}$ and $Y = \{y_1, \dots, y_n\}$, Pearson's correlation is

$$r(X, Y) = \frac{\sum_{i=1}^{n} (x_i - \bar{x})(y_i - \bar{y})}{\sqrt{\sum_{i=1}^{n} (x_i - \bar{x})^2} \sqrt{\sum_{i=1}^{n} (y_i - \bar{y})^2}}\,, \tag{30}$$

where $\bar{x}$ and $\bar{y}$ are the mean of the variables $\{x_1, \dots, x_n\}$ and $\{y_1, \dots, y_n\}$, respectively. Pearson's correlation is bounded between -1 (complete negative correlation) and 1 (complete positive correlation). To compute the statistical significance (p-value of the result), we first compute

$$t = r(X, Y) \frac{\sqrt{n-2}}{\sqrt{1 - r(X, Y)^2}}\,, \tag{31}$$

which follows Student's t distribution under the null hypothesis that X and Y are uncorrelated. The probability of obtaining Pearson correlation $r \geq |r(X, Y)|$ by chance can then be obtained by evaluating the cumulative distribution function.

Mann-Whitney's U statistic is a non-parametric measure of the difference in distribution of two sets of ordinal variables (Mann and Whitney, 1947). Let us again consider two collections of variables, but this time with potentially different numbers of elements $X = \{x_1, \dots, x_m\}$, $Y = \{y_1, \dots, y_n\}$. To compute U, we compute the rank of each of the variables in the full set $\{x_1, \dots, x_m, y_1, \dots, y_n\}$ from 1 (smallest) to $n + m$ (largest). If two or more variables share the same value, they are each assigned the average of the rank of all variables with the same value. For example, the ranks corresponding to the set of variables $\{0, 0.5, 0.5, 0.5, 1\}$ would be $\{1, 3, 3, 3, 5\}$. Then we calculate

$$U = n\,m + \frac{m(m+1)}{2} - \sum_{i=1}^{m} \text{rank}(x_i)\,. \tag{32}$$

Similarly one could compute a value U' in the same way as above, but with X and Y (and the corresponding number of variables in each) exchanged; these values are linked by the relation $U + U' = n\,m$. Following convention we report the smaller of the U values. The smaller the U value, the greater the difference in the ranks of the variables belonging to X and Y. When the number of data points is large, the distribution for U under the null hypothesis that variables in X and Y are identically distributed approximately follows the normal distribution, allowing for the computation of the statistical significance of a certain value of U. The exact distribution of U can also be computed when the number of samples is not too large, as in the cases considered here.

Supplementary Figures

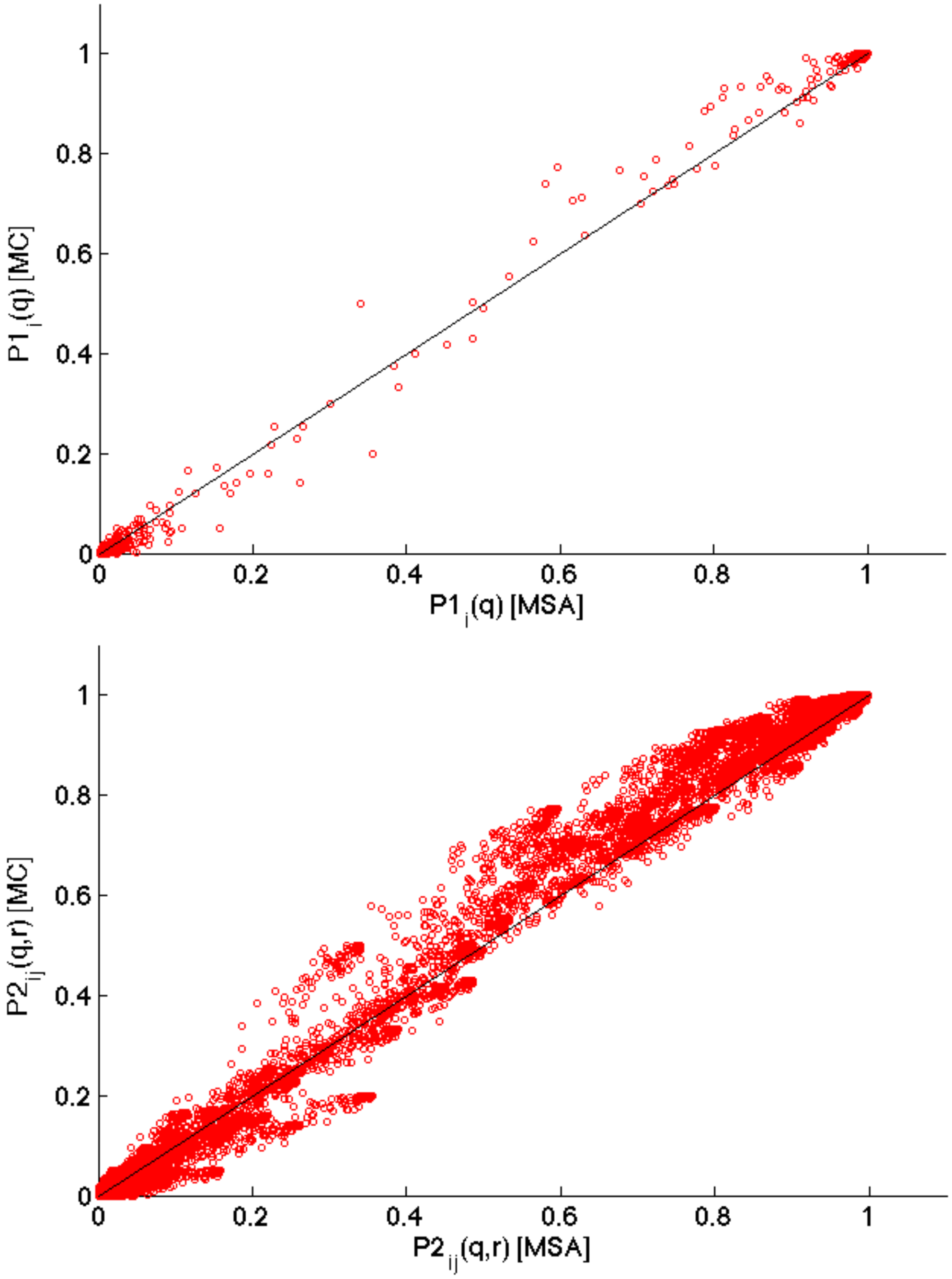

Figure 1. Comparison of the p17 one and two-position amino acid frequencies observed in the MSA with those computed from the final Potts model by performing 2×10^5 steps of MCMC sampling.

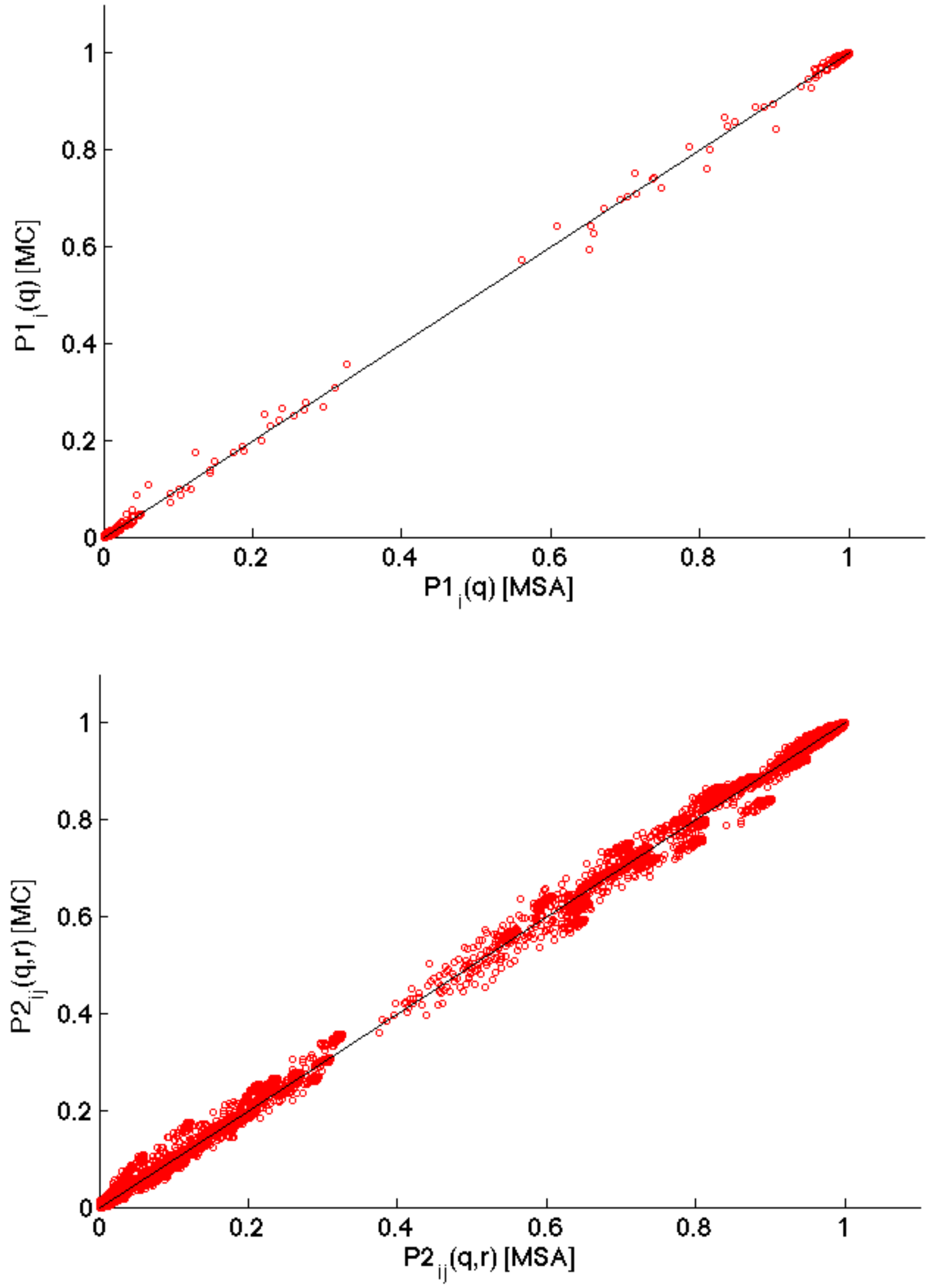

Figure 2. Comparison of the p24 one and two-position amino acid frequencies observed in the MSA with those computed from the final Potts model by performing 10^6 steps of MCMC sampling.

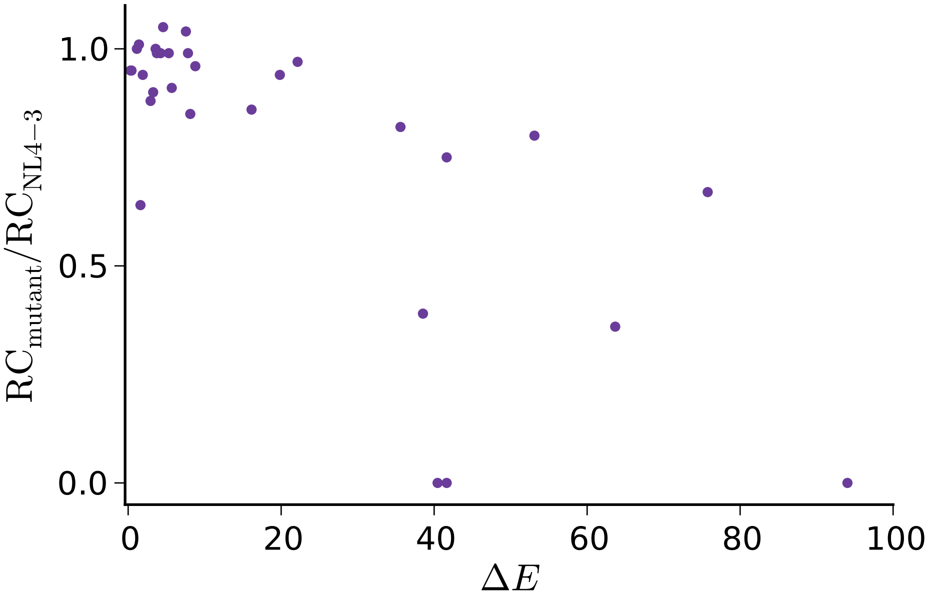

Figure 3: Scatter plot of the energy difference ΔE computed by the unregularized Ising model (Ferguson *et al.*, 2013) and the corresponding RC ratio for each of the experimentally tested mutants. Mutants with $E = \infty$ are not shown ($n = 13$).

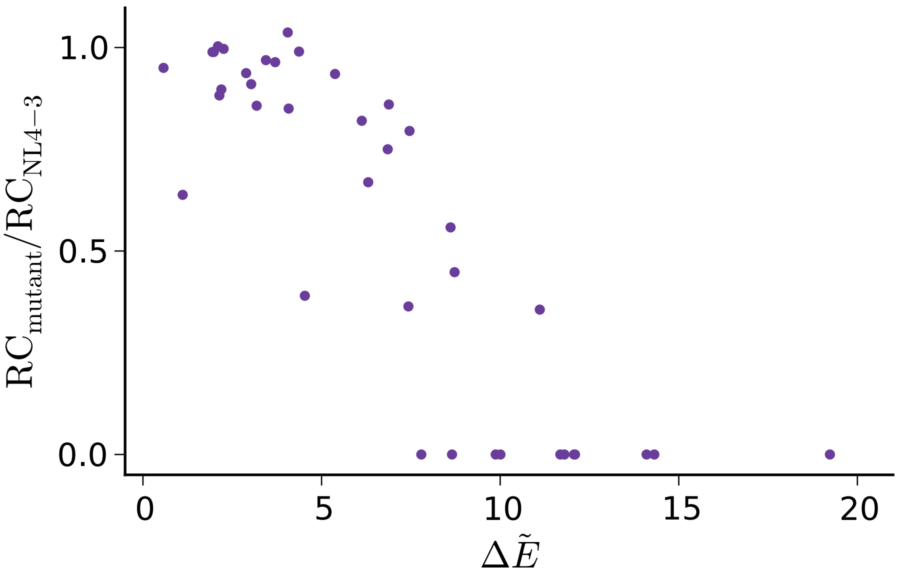

Figure 4: Scatter plot of the mutation-corrected energy difference $\Delta \tilde{E}$ between mutant and the NL4-3 reference (Eqn. 29) and the corresponding RC ratio (Eqn. 22) for each of the experimentally tested mutants.

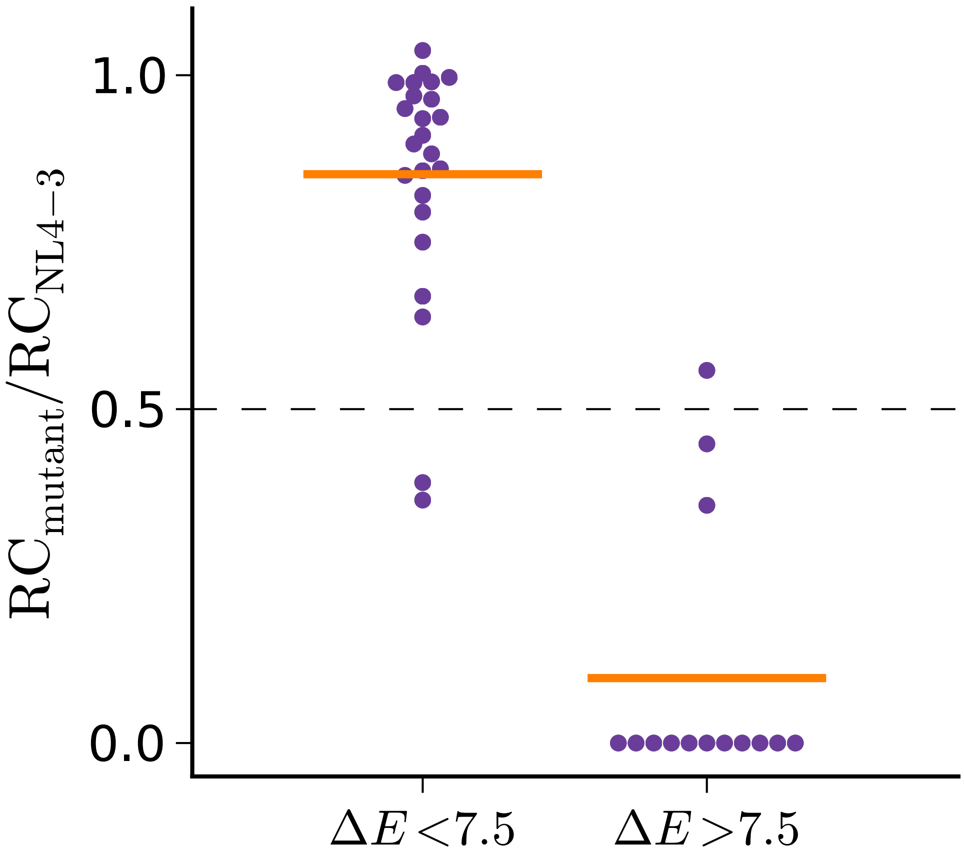

Figure 5: Plot of mutant RC ratio versus mutation-corrected energy difference $\Delta\tilde{E}$ between the mutant and the NL4-3 reference (Eqn. 29), for each of the experimentally tested p24 mutants. Mutants with $\Delta\tilde{E} < 7.5$ are predicted to be *fit* (RC ratio > 0.5), while those with $\Delta\tilde{E} > 7.5$ are predicted to be *unfit* (RC ratio ≤ 0.5).

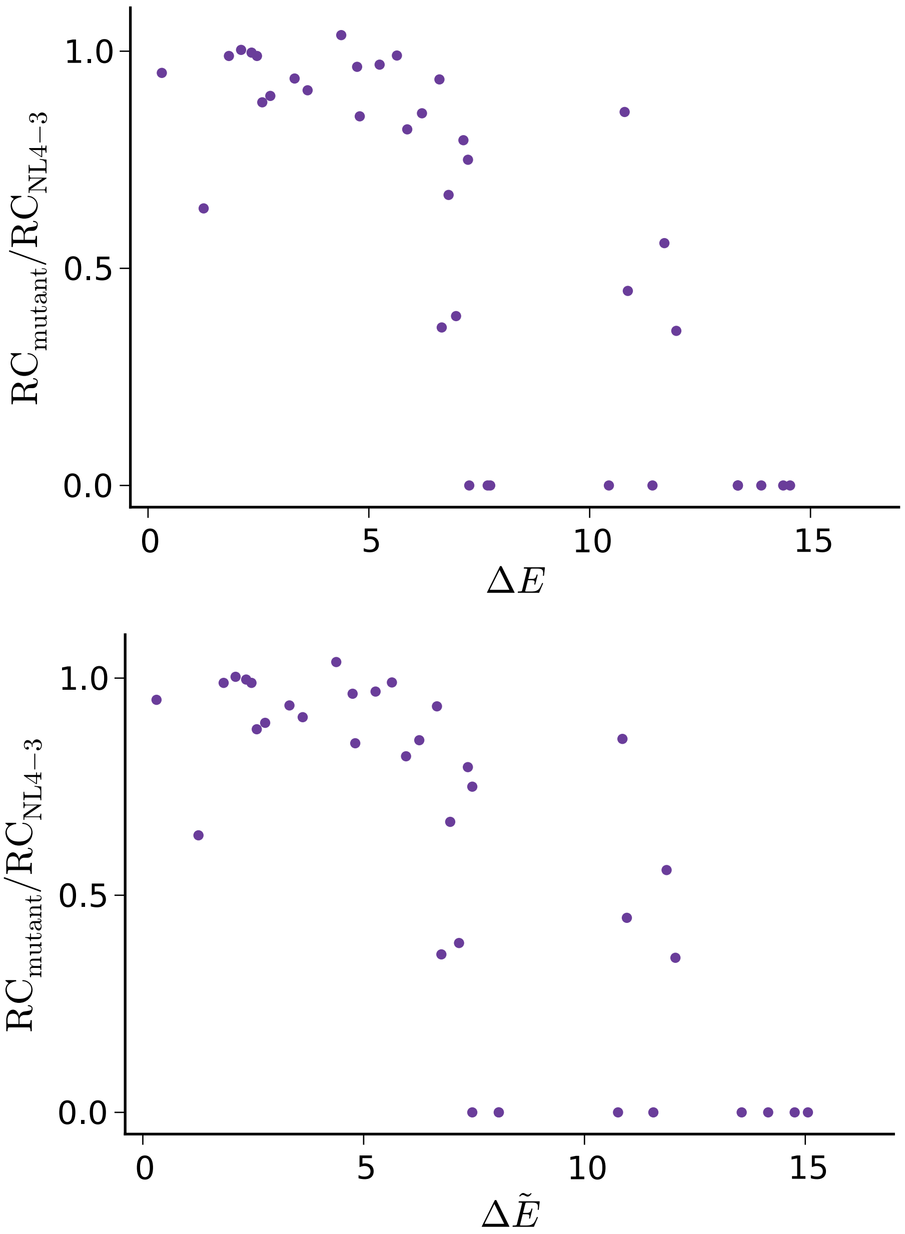

Figure 6: Scatter plot of the Potts model energy difference ΔE (*top*) and mutation-corrected energy difference $\Delta \tilde{E}$ (*top*) between mutants and the NL4-3 reference (Eqn. 29) and the corresponding RC ratio (Eqn. 22) for each of the experimentally tested p24 mutants. Note the small size of the correction in this case.

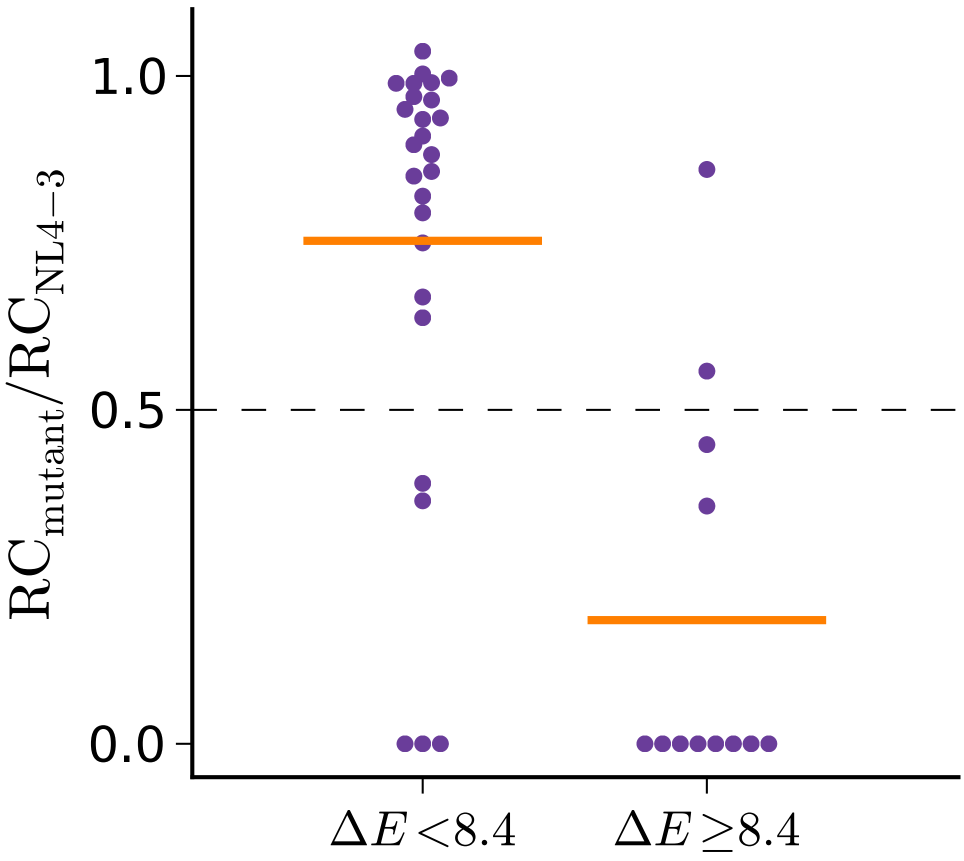

Figure 7: Plot of mutant RC ratio versus energy difference ΔE between the mutant and wild-type computed with a Potts model, for each of the experimentally tested p24 mutants. Mutants with $\Delta E < 8.4$ are predicted to be *fit* (RC ratio > 0.5), while those with $\Delta E > 8.4$ are predicted to be *unfit* (RC ratio ≤ 0.5).

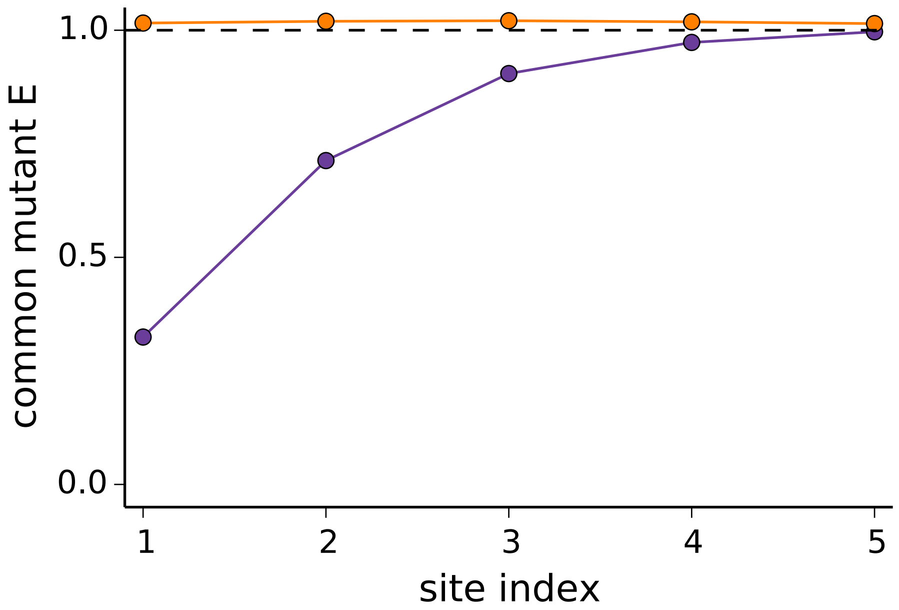

Figure 8: Plot of inferred common single mutant energies in the toy model, using an Ising model (*purple*) and a Potts model (*orange*), versus the true value (*dashed*). The Ising model underestimates the energy of the most common single mutant at sites where other mutants occur with similar frequency (sites with low indices). The Potts model can infer these energies accurately.

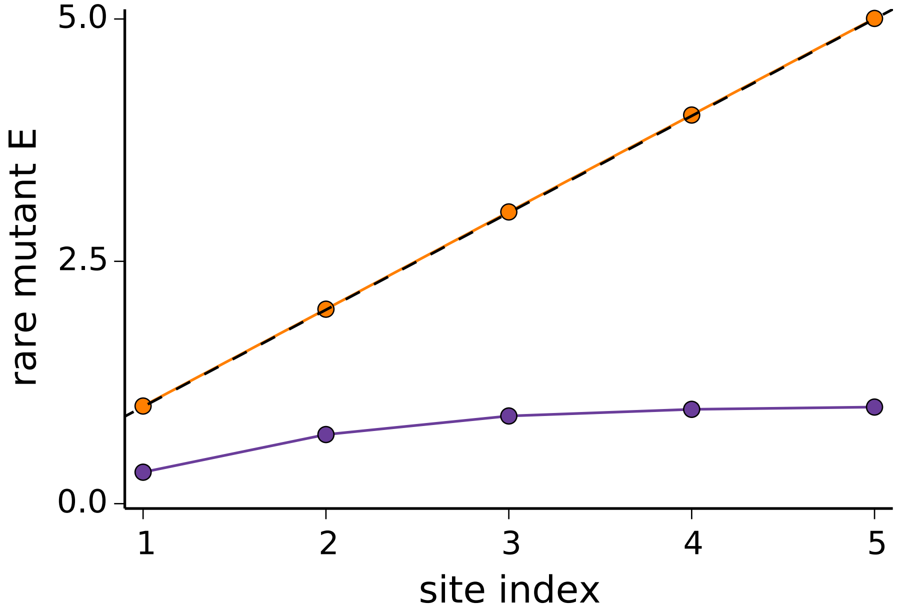

Figure 9: Plot of inferred rare single mutant energies in the toy model, using an Ising model (*purple*) and a Potts model (*orange*), versus the true value (*dashed*). The Ising model severely underestimates the energy of rare single mutants at sites where other mutants occur with far greater frequency (sites with high indices). The Potts model can infer these energies accurately.

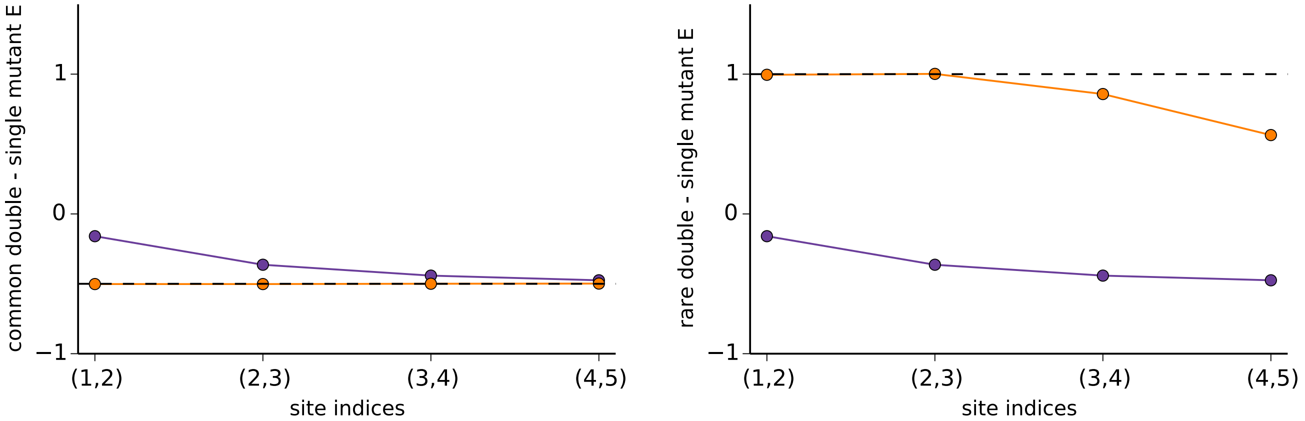

Figure 10: Plot of the double mutant energy minus the sum of the single mutant energies at each pair of nearest neighbor sites, using an inferred Ising model (*purple*) and a Potts model (*orange*), against the true values (*dashed*). Here we only consider cases where both mutants are to the common amino acid variant (*left*) or to the rare variant (*right*). The Ising model gives the same energy difference in both cases. The Ising energy difference is closer to the true energy difference when both mutations are to the most common variant. The Potts model accurately reproduces the energy difference in both cases, though its accuracy suffers slightly when attempting to predict a strong negative coupling between the rarest mutants, the rare amino acid variants at sites $(4, 5)$.

Supporting References

Barton, J. and Cocco, S. (2013). Ising models for neural activity inferred via selective cluster expansion: structural and coding properties. *Journal of Statistical Mechanics: Theory and Experiment*, 2013(03):P03002.

Binder, K. and Young, A. P. (1986). Spin glasses: Experimental facts, theoretical concepts, and open questions. *Reviews of Modern physics*, 58(4):801.

Cocco, S. and Monasson, R. (2011). Adaptive cluster Expansion for inferring Boltzmann machines with noisy data. *Physical Review Letters*, 106(9).

Cocco, S. and Monasson, R. (2012). Adaptive cluster expansion for the inverse Ising problem: convergence, algorithm and tests. *Journal of Statistical Physics*, 147(2):252–314.

DeGroot, M. H. and Schervish, M. J. (2002). *Probability and statistics*. Addison Wesley.

Ferguson, A. L., Mann, J. K., Omarjee, S., Ndungu, T., Walker, B. D., and Chakraborty, A. K. (2013). Translating hiv sequences into quantitative fitness landscapes predicts viral vulnerabilities for rational immunogen design. *Immunity*, 38(3):606–617.

Frenkel, D. and Smit, B. (2002). *Understanding molecular simulation: from algorithms to applications*. Elsevier (formerly published by Academic Press).

Hastie, T., Tibshirani, R., and Friedman, J. (2009). *The Elements of Statistical Learning*. Data Mining, Inference, and Prediction, Second Edition. Springer.

Jaynes, E. T. (1957). Information theory and statistical mechanics. *Physical review*, 106(4):620.

Jaynes, E. T. (1982). On the rationale of maximum-entropy methods. *Proceedings of the IEEE*, 70(9):939–952.

Leitner, T., Korber, B., Daniels, M., Calef, C., and Foley, B. (2005). HIV-1 subtype and circulating recombinant form (CRF) reference sequences, 2005. *HIV sequence compendium*, 2005:41–48.

Lezon, T. R., Banavar, J. R., Cieplak, M., Maritan, A., and Fedoroff, N. V. (2006). Using the principle of entropy maximization to infer genetic interaction networks from gene expression patterns. *Proceedings of the National Academy of Sciences*, 103(50):19033–19038.

Mann, H. B. and Whitney, D. R. (1947). On a test of whether one of two random variables is stochastically larger than the other. *The Annals of Mathematical Statistics*, 18(1):50–60.

Mora, T. and Bialek, W. (2011). Are biological systems poised at criticality? *Journal of Statistical Physics*, 144(2):268–302.

Morcos, F., Pagnani, A., Lunt, B., Bertolino, A., Marks, D. S., Sander, C., Zecchina, R., Onuchic, J. N., Hwa, T., and Weigt, M. (2011). Direct-coupling analysis of residue coevolution captures native contacts across many protein families. *Proceedings of the National Academy of Sciences*, 108(49):E1293–E1301.

Nocedal, J. and Wright, S. J. (1999). *Numerical Optimization.* Springer Verlag.

Peterson, W. W., Birdsall, T. G., and Fox, W. (1954). The theory of signal detectability. *Transactions of the IRE Professional Group on Information Theory*, 4(4):171–212.

R Core Team (2012). *R: A Language and Environment for Statistical Computing.* R Foundation for Statistical Computing, Vienna, Austria. ISBN 3-900051-07-0.

Roudi, Y., Aurell, E., and Hertz, J. A. (2009a). Statistical physics of pairwise probability models. *Frontiers in computational neuroscience*, 3.

Roudi, Y., Tyrcha, J., and Hertz, J. (2009b). Ising model for neural data: Model quality and approximate methods for extracting functional connectivity. *Physical Review E*, 79(5):051915.

Sella, G. and Hirsh, A. E. (2005). The application of statistical physics to evolutionary biology. *Proceedings of the National Academy of Sciences of the United States of America*, 102(27):9541–9546.

Shekhar, K., Ruberman, C. F., Ferguson, A. L., Barton, J. P., Kardar, M., and Chakraborty, A. K. (2013). Spin models inferred from patient-derived viral sequence data faithfully describe hiv fitness landscapes. *Physical Review E*, 88(6):062705.

Sivia, D. S. (1996). *Data Analysis.: A Bayesian Tutorial.* Oxford University Press.

Weigt, M., White, R. A., Szurmant, H., Hoch, J. A., and Hwa, T. (2009). Identification of direct residue contacts in protein–protein interaction by message passing. *Proceedings of the National Academy of Sciences*, 106(1):67–72.

Wright, S. (1932). The roles of mutation, inbreeding, crossbreeding and selection in evolution. In *Proceedings of the sixth international congress on genetics*, volume 1, pages 356–366.

Wu, F.-Y. (1982). The potts model. *Reviews of modern physics*, 54(1):235.
